# Predictors of knowledge about tuberculosis: results from SANHANES I, a national, cross-sectional household survey in South Africa

**DOI:** 10.1186/s12889-016-2951-y

**Published:** 2016-03-18

**Authors:** Pamela Naidoo, Leickness Simbayi, Demetre Labadarios, Yoliswa Ntsepe, Nwabisa Bikitsha, Gadija Khan, Ronel Sewpaul, Sizulu Moyo, Thomas Rehle

**Affiliations:** Population Health, Health Systems and Innovation Research Programme, Human Sciences Research Council, Pretoria, Durban and Cape Town, South Africa, Private Bag X9182, Cape Town, 8000 South Africa; Department of Psychology, University of the Western Cape, Cape Town, South Africa; HIV, AIDS, Sexually Transmitted Diseases and Tuberculosis (HAST) Research Programme, Human Sciences Research Council, Pretoria, Durban and Cape Town, Cape Town, South Africa; Department of Psychiatry and Mental Health, University of Cape Town, Cape Town, South Africa; Centre for Infectious Disease, Epidemiology and Research, School of Public Health and Family Medicine, University of Cape Town, Cape Town, South Africa

**Keywords:** Cross-sectional national survey, Adult participants 18–64 years old, Tuberculosis (TB) knowledge, Social determinants of TB, TB/HIV co-existence, High burden country

## Abstract

**Background:**

South Africa is one of the 22 high tuberculosis burden countries that contribute 80 % of the global tuberculosis cases. Tuberculosis is infectious and due to its rapid and easy transmission route poses a threat to population health. Considering the importance of social and psychological factors in influencing health outcomes, appraising knowledge and awareness of tuberculosis, remain vital for effective tuberculosis control. The main aim of this study was to investigate the factors that predict knowledge about tuberculosis among 18–64 year old adults in South Africa.

**Methods:**

A cross-sectional survey method was used. Multi-stage disproportionate, stratified cluster sampling was used to select households within enumeration areas stratified by province and locality type. Based on the Human Sciences Research Council 2007 master sample, 500 Enumerator Areas representative of the socio-demographic profile of South Africa were identified and a random sample of 20 households was randomly selected from each Enumerator Area, yielding an overall sample of 10 000 households. The tuberculosis module contained in the South African National Health And Nutrition Examination Survey I was the only module that examined the social determinants of an infectious disease. This module was questionnaire-based with no biomarkers obtained to screen for the presence of tuberculosis disease among the participants. Data was collected by administering a researcher developed individual level questionnaire. Simple and multiple linear regression was used to determine the independent variables associated with tuberculosis knowledge.

**Results:**

Half the sample (52.6 %) was female and the majority of the respondents were black African (76.5 %). More than two thirds (68.0 %) resided in urban areas, 56.9 % did not complete high school and half were not in formal employment. Significant predictors of tuberculosis knowledge were race, sex, completion of high school, being in employment, having a diagnosis of the disease in ones’ life-time and learning about tuberculosis from television, brochures, health workers, and teachers.

**Conclusions:**

To reduce the burden of tuberculosis in South Africa, media campaigns targeting both rural and urban communities should include conveying accurate information about the disease. Policy makers should also address structural barriers that vulnerable communities face.

**Electronic supplementary material:**

The online version of this article (doi:10.1186/s12889-016-2951-y) contains supplementary material, which is available to authorized users.

## Background

Tuberculosis (TB) disease is a global public health problem with a third of the world’s population infected with mycobacterium tuberculosis [1]. The disease is present in all regions of the world with a quarter of the estimated 9 million people who developed TB in 2013 living in the African region [2]. South Africa (SA) ranks as the sixth highest in generating new cases of TB in the world, and is among the 22 high TB-burden countries that contribute about 80 % of the global cases of TB [2]. The country’s TB burden can, in part, be attributed to the conditions of poverty that remain prevalent in many parts of the country. According to Lutge et al. such conditions favour the transmission of the disease linking poverty to a greater risk of infection, poorer health outcomes and lower levels of health seeking behaviour [3].

TB is one of the high burden diseases in SA and due to its infectious nature, rapid and easy transmission route, poses a threat to population health in the country. In addition there has been a steady increase in the burden of the drug resistant TB which poses a threat to TB control interventions [4–6]. The high rate of human immunodeficiency virus (HIV) and TB co-infection which is responsible for 25 % of all TB-HIV co-infections in the world [7] has also placed demands on the health-care sector in SA. Results of the 2012 South African National HIV Prevalence, Incidence and Behaviour Survey showed that the common co-existence of TB and HIV is also recognised by the general population who when asked if people with TB should be tested for HIV, 81 % agreed [8]. Thus, prevention and treatment of TB is one of the key priorities of the National Department of Health [9]. TB is a curable disease which requires strict and consistent adherence to the prescribed treatment regimens.

### Biomedical versus socio-psychological research on TB

Whilst biomedical research on TB has been prolific, research on the social and psychological drivers of the disease has been neglected despite the acknowledgement of the importance of the social determinants of health in general, and TB in particular [10–12]. Biomedical research has largely focussed on examining disease pathways, developing rapid diagnostic techniques and effective medicines that can treat and cure those who are diagnosed with TB. Despite these medical advances and the slight decline observed in recent years, TB prevalence in SA remains unacceptably high [2, 4]. The role of social and psychological factors as determinants of acquiring TB infection and in curing the disease has been under-estimated and understudied.

### Knowledge about TB as an infectious disease

Sreeramareddy et al. state that delays in treatment seeking may be due to the pervasive lack of awareness of the symptoms of TB [1]. TB control programs have thus recognized the importance of providing information, education and communication aimed at improving the knowledge about TB and influencing change in health-care seeking behaviour among both TB patients and the general public [1]. Among the factors that make it difficult for people to effectively use TB prevention information is that TB literature is mostly presented from biomedical perspectives. Studies show that it is imperative to understand the lay perceptions that determine health- seeking behaviour [13].

Factors such as poor public awareness about TB, in addition to a limited engagement of communities and non-governmental health organizations, have been identified as a challenge that impedes TB control [14]. Therefore, having appropriate knowledge and awareness of TB and, in particular, knowing that the disease is curable even in the presence of HIV remains a vital message required for effective TB control in SA [15]. Knowledge about the duration of treatment is also very important for successful treatment and cure [2]. Health messages about TB diagnosis, treatment and cure are particularly important when TB patients decide to utilize health services, seek a diagnosis and adhere to treatment [16, 17]. Knowledge and awareness of the infectiousness of TB as a disease is the basis for individuals taking protective measures to avoid becoming infected, or transmitting it to others for those with active disease. Prolonged diagnostic and treatment delays, particularly in settings experiencing concomitant HIV and TB epidemics, may undermine the global TB control efforts [17].

Examining the social and psychological factors associated with TB onset is important given the fact that knowing these factors can help health practitioners design behaviour change interventions for disease prevention, treatment adherence, and access to TB health care services [18]. This study sought to address the gap between biomedical and social research by investigating the level of knowledge of TB at a population level among adults. The study was based on the data obtained from the South African National Health and Nutrition Examination Survey (SANHANES I) [19] which included a module, that ascertained the Knowledge, Attitude, Belief and Practice (KABP) of TB.

Knowledge of TB has been defined and measured in various ways in various studies, such as those by Ottmani et al., Abebe et al., and Obuku et al. [20–22]. In this study knowledge of TB was defined and measured by the study participants’ knowledge of the following: prevention of TB; TB symptoms; TB transmission routes; TB treatment; and TB and HIV co-existence. The main aim of this study was to investigate the factors that predict knowledge about TB as an infectious disease among adults (18–64 years) in SA.

## Methods

### Study design, sample and procedures

SANHANES I was a national survey that assessed defined aspects of the health and nutritional status of South Africans primarily with respect to the prevalence of selected non-communicable diseases and their risk factors [19]. The survey also assessed the knowledge, attitudes and behaviour of South Africans of one high burden infectious disease, namely TB.

Based on the HSRC 2007 master sample, 500 Enumerator Areas (EAs) representative of the socio-demographic profile of South Africa (SA) were identified and a random sample of 20 visiting points (households) were randomly selected from each EA, yielding an overall sample of 10 000 visiting points. EAs were sampled with probability proportional to the size of the EA using the 2001 census estimate of the number of visiting points (VPs) in the EA database as a measure of size (MOS).

In the sampled visiting point, all household members were eligible to participate in the survey (see Additional file 1). The privacy of household members was ensured during their participation. In instances where the dwelling was too small and lacked a private space to conduct an interview, participants were interviewed in private outside their home but on their property.

The survey obtained socio-demographic and disease specific questionnaire-based data through interviews at the household level (see Additional file 2). Clinical examinations including health measurements, a selection of clinical tests as well as the collection of a blood sample were also conducted. Life-time prevalence of TB diagnosis was, however, obtained through self-report and not biomedical data. There was also no diagnostic testing for HIV. Interviews were conducted by trained fieldworkers and the biomarker and anthropometric data was collected by health practitioners, which included medical doctors and certified nurses. The field staff and health practitioners attended a 5-day training workshop on how to complete the various sections of the questionnaire. To ensure standardization the health practitioners also went through the clinical examination protocol during the training. The questionnaires were translated from English to all national languages and then back translated. Field staff was matched for language spoken in the various regions in SA. All the questionnaires were pre-tested before the survey.

### Data collection relevant to the TB module of SANHANES I

#### Knowledge questionnaire

Items specific to the knowledge component that were used for this study comprised of questions pertaining to the knowledge of the symptoms of TB (based on the World Health Organization’s 8-item TB symptom knowledge brief questionnaire) [23], TB transmission, TB cure, prevention of TB, and TB/HIV co-existence. Respondents were also asked if they had ever been *diagnosed with TB*, and where they *had initially learned* about TB, with multiple response options for the sources possible from the following: newspapers and magazines, radio, television (TV), brochures, health workers, family, friends, colleagues, and teachers. Each reported option was used as a separate variable corresponding to each information source, e.g. TV, radio, teachers. (Please note this when making inferences on results pertaining to the information sources of TB).

Respondents were also asked their o*pinions on the three most effective sources of information on TB from the a predefined list* (newspapers and magazines radio, television, billboards, brochures, posters and other printed materials, health workers, family, friends, neighbours and colleagues, religious leaders, teachers or other sources).

### Measures

The measures mentioned below, were obtained from the socio-demographic questionnaire and from the TB-specific module. Age group, sex, race, urban/rural status, completion of high school, employment, annual income category, having ever been diagnosed with TB and sources of information from which the respondents initially learned about TB were used as the independent variables.

*Race* was reported as per Statistics South Africa [24]. Race is an important stratification variable within the South African context given the fact that black African, people of Indian origin and those of mixed racial heritage have been historically disadvantaged. By utilizing the racial categories of black African, Indian, mixed ancestry, and white, a health researcher is able to report on health inequalities, advantages and disadvantages in each of these communities.

*Urban and rural status* were determined based on the urban/rural status of the enumerator area in which the participant’s household was located, and this information was available from the census master sample [24].

*Completion of high school was used as an indicator of education level*: participants who reported that their highest level of education completed was grade 12/matric or equivalent and those with tertiary education were categorised as having completed high school.

*Employment*: Participants who were self-employed or employed either on a full-time or a part time basis were categorised as being employed, while those who were unemployed regardless of whether they were or were not seeking employment, those who were homemakers, students, retirees or were sick, disabled or unable to work were categorised as unemployed.

*Annual income*: Participants were asked their individual annual income amount. Based on the response range and distribution, responses were classified into no income; <=R 9600 per annum (approx. $800); R9601 – R38 400 (approx. $800 – S3 200) and over R38 400.

*Having ever been diagnosed with TB* was based on respondents’ self- report.

### Ethics

The study received approval from the Research Ethics Committee (REC) of the Human Sciences Research Council (HSRC) (REC number: 6/16/11/11). The HSRC’s REC is aligned with the Declaration of Helsinki and has Federal-wide Assurance (FWA) for the Protection of Human Subjects accreditation with the USA’s Department of Health and Human Services (DHHS). A full consenting process was applied in respect of all participants. Consent to use the survey data was also obtained.

### Data analysis

Data were analysed using STATA 12 (Stata Corp., College Station, TX). Data were weighted to account for unequal sampling probabilities and to benchmark in line with the 2012 South African mid-year population estimates. The primary outcome variable, *composite knowledge about TB*, was constructed based on the responses to the 6 individual knowledge items described below.

1. *Knowledge of symptoms*: respondents scored a 1 if 3 of the 6 essential symptoms of TB which were listed in the response options (according to WHO list of TB symptoms) were correctly identified, and scored 0 if they listed < 3 symptoms.
2. *Knowledge of transmission of TB*: respondents scored 1 if the primary transmission method of TB i.e. through the air when a person sneezes or coughs, was correctly identified, without listing any misconceptions and scored 0 for any other response.
3. *Knowledge of prevention*: respondents scored 1 if the main prevention method i.e. covering the mouth when coughing or sneezing was correctly identified and scored 0 for any other responses.
4. *Knowledge that TB is curable and correct knowledge of treatment without misconceptions*: respondents scored 1 if they reported that TB can be cured and that it can be cured by specific drugs given by a health centre or by (Directly Observed Treatment Short course (DOTS), and scored 0 if they reported either that they thought TB could not be cured or that it can be cured but listed the incorrect treatment methods.
5. *Knowledge that TB patients should be tested for HIV*: participants scored 1 if they reported this and scored 0 for any other response.
6. *Knowledge that people with HIV are more likely to develop TB*: participants scored 1 if they reported this and scored 0 for any other response.

All 6 TB knowledge questions included a ‘Don’t know’ option, which was coded as 0, which indicated that a participant did not have correct knowledge about that item. The scores of the 6 items were summed to produce a composite knowledge score ranging from 0 to 6.

Simple and multiple linear regression analysis was used to test for associations between the level of composite TB knowledge, and socio-demographic characteristics, first source of information about TB and history of ever being diagnosed with TB. Variables that were statistically significant in the simple regression analysis (*p* <0.05) were included in the multiple regression. (The multivariable analysis controlled for age and sex). The prevalence of correct knowledge for each of the six individual knowledge items as well as the mean composite TB knowledge score is presented by socio-demographic variables, life time history of TB diagnosis and first source of information about TB. Estimates were considered to be significantly different if their 95 % confidence intervals did not overlap.

## Results

### Characteristics of the sample

Table 1 provides the socio-demographic characteristics, self-reported history of TB diagnosis and the initial source of information about TB of the 5945 participants aged between 18 and 64 years who completed the interviews and answered questions relating to TB and socio-economic status. The mean age was 38.4 years (SD = 13.2). The study sample comprised 52.6 % females and the majority of the respondents were black African (76.5 %). More than two thirds (68.0 %) resided in urban areas, 56.9 % did not complete high school and half (50.4 %) were not in formal employment. Over a third (34.6 %) reported having no individual income, 11.4 % received an annual income of under R 9 600 (approx. $800), and the remaining 54.0 % received over R 9 600 per annum. Seven percent (7.0 %) reported having ever been diagnosed with TB in their life-time. When respondents were asked through what methods they had initially learned about TB (multiple responses were permissible), the most commonly reported responses were via radio (49.2 %), television (45.8 %), and through health workers (38.2 %). Almost a quarter (23.9 %) of respondents first learnt about TB from teachers, 23.2 % in newspapers and magazines, 17.7 % from friends, family, colleagues or neighbours and 8.9 % from brochures. A tenth (10.6 %) of the respondents reported other sources of information, which included information from billboards, religious leaders, at work, in prison and knowing someone who was diagnosed with TB. A further 0.7 % reported not knowing about or having never heard of TB.

**Table 1:** Demographic and socio-economic characteristics, TB diagnosis and first source of information on TB among adults aged 18–64 years

	%	95 % CI	n
Total	100.0		5945
Age (Years)			
18 to 24	17.7	[16.4–19.2]	1168
25 to 34	28.0	[26.0–30.2]	1405
35 to 44	24.6	[22.7–26.6]	1283
45 to 54	18.4	[16.3–20.6]	1218
55 to 64	11.2	[9.8–12.8]	871
Sex			
Female	52.6	[50.7–54.5]	3509
Male	47.4	[45.5–49.3]	2436
Race			
African	76.5	[71.8–80.6]	3824
White	10.8	[7.5–15.3]	312
Coloured	10.6	[8.9–12.6]	1394
Indian	2.1	[1.3–3.4]	415
Urban / Rural			
Rural	32.0	[27.3–37.1]	1850
Urban	68.0	[62.9–72.7]	4095
Completion of high school/equivalent			
Did not complete high school	56.9	[53.9–59.8]	3774
Completed high school (Grade 12 or higher)	43.1	[40.2–46.1]	2171
Employment Status			
Unemployed	50.4	[47.4–53.4]	3183
Employed	49.6	[46.6–52.6]	2762
Annual income			
No income	34.6	[32.1–37.1]	2118
<–R9 600	11.4	[10.0–13.1]	682
R9 601–R38 400	28.8	[26.6–31.2]	1896
>–R38 401	25.2	[22.2–28.4]	1249
Ever been diagnosed with TB	7.0	[5.9–8.2]	458
Source of information on TB: Where did you first learn about TB?			
Newspapers and magazines	23.2	[20.7–25.9]	1377
Radio	49.2	[46.1–52.3]	2849
Television	45.8	[42.6–49.0]	2588
Brochures, pamphlets or other printed materials	8.9	[7.4–10.6]	495
Health workers	38.2	[35.7–40.7]	2235
Family, friends, colleagues, neighbours	17.7	[15.9–19.7]	1090
Teachers	23.9	[21.9–26.1]	1508
Other sources of information	10.6	[9.1–12.4]	657
Never heard of or don’t know about TB	0.7	[0.4–1.0]	39

### Correct knowledge on individual knowledge items (See Additional file 3)

#### Knowledge of TB symptoms

Overall, 21.5 % of participants correctly identified any three of the six key symptoms of TB from the WHO standard list of TB symptoms. Knowledge of symptoms did not differ significantly by sex, age group, rural/urban residential status, education status, employment, annual income, and personal history of TB diagnosis. White adults (34.1 % [25.5–44.0]) had significantly higher knowledge of symptoms than those of mixed race (16.5 % [12.8–21.0]) and black Africans (20.4 % [17.8 – 23.3]) adults. Knowledge of symptoms was significantly higher among those who initially learnt about TB from newspapers and magazines (28.9 % [24.6–33.7]) than those who did not (19.2 % [16.8–21.9]). In addition, for the following channels of information, knowledge of symptoms was significantly higher among those who were exposed to TB information from these sources compared to those who were not: radio (27.2 % [23.7–31.0] vs 15.9 % [13.3–18.9]), TV (27.7 % [24.0–31.7] vs 16.2 % [13.7–19.1]), brochures (37.3 % [29.2–46.2] vs 19.9 % [17.4–22.7]), and health workers (28.8 % [25.3–32.5] vs 17.0 % [14.5–19.8]).

#### Knowledge of TB transmission

Almost two thirds (63.0 %) correctly identified the primary transmission route of TB, i.e. through the air when a person sneezes or coughs, without any misconceptions. Knowledge of transmission did not differ significantly by sex, age group, race, rural/urban residential status, education (completion of high school), employment, annual income, history of TB diagnosis and each of the sources of information on TB.

#### Knowledge of the prevention of TB onset

Overall, 79.0 % correctly identified the main TB prevention method i.e. covering the mouth when coughing or sneezing. Knowledge of prevention did not differ significantly by sex, age group, race, rural/urban residential status, education (completion of high school), and TB diagnosis. Employed adults (82.5 % [79.9–84.7]) had significantly higher prevalence of knowledge of prevention than unemployed adults (75.7 % [72.8–78.3]). The prevalence of correct knowledge of prevention was also significantly higher among those receiving an annual income of more than R 38400 (84.6 % [81.0–87.6]) than those receiving no income (76.8 % [73.2–80.0]). Knowledge of prevention was significantly higher among those who were first exposed to TB information from these channels/sources compared to those who were not: radio (83.6 % [80.3–86.4] vs 74.7 % [71.8–77.3]), television (86.1 % [83.3–88.5] vs 73.0 % [70.0–75.9]), brochures (90.5 % [86.6–93.3] vs 77.9 % [75.7–80.0]), and health workers (83.7 % [81.1–86.0] vs 76.1 % [73.5–78.6]). Contrastingly, respondents who were exposed to TB information from family, friends, neighbours and colleagues had lower levels of correct knowledge of prevention that those who did not report learning about TB from these people (71.6 % [65.9–76.7] vs 80.6 % [78.5–82.6]).

#### Knowledge of the curability of TB disease and the correct TB treatment method

A large proportion (83.9 %) displayed correct knowledge that TB is curable and knew about the correct treatment without any misconceptions i.e. that TB can be cured by specific drugs given by a health centre or by the Directly Observed Treatment Short-Course (DOTS). Knowledge of curability and treatment did not differ significantly by sex, age group, rural/urban residential status, , employment and annual income. Adults of mixed race (90.5 % [87.8–92.6]) had significantly higher levels of correct knowledge about treatment and curability than black African (82.9 % [80.3–85.2]) adults. Significantly more adults who had completed high school (87.0 % [84.1 – 89.5]) had correct knowledge about treatment and curability than those who had not completed high school (81.5 % [78.8 – 83.9]). Those who had previously been diagnosed with TB (90.9 % [86.6–93.9]) had significantly higher levels of knowledge of curability and treatment than those who had never had TB (83.3 % [80.9–85.5]). Knowledge of curability and treatment was significantly higher among those who were initially exposed to TB information from health workers (88.7 % [85.9–90.9] vs 80.9 % [77.9–83.6]) compared to those who did not receive information from health workers.

#### Knowledge that TB patients should be tested for HIV

Overall, 83.1 % correctly responded that TB patients should be tested for HIV, with no significant variation in the proportion by sex, age group, rural/urban residential status, annual income and having ever been diagnosed with TB. Significantly more adults of mixed race (89.2 % [85.4–92.2]) than black African adults (81.8 % [79.4–83.9]) knew that TB patients should be tested for HIV. Knowledge that TB patients should be tested for HIV was significantly higher among those who had completed high school (88.3 % [85.6–90.6]) than those who did not (79.2 % [76.6–81.5]), and significantly higher among the employed (86.0 % [83.3–88.3]) than the unemployed (80.3 % [78.0–82.4]). For each of the sources of information on TB, that is, the media sources and information from other people, the prevalence of knowing that TB patients should be tested for HIV did not vary significantly for those exposed to each source of information versus those who were not.

#### Knowledge that people with HIV are more likely to develop TB

Over three quarters of participants correctly responded that people with HIV are more likely to develop TB, with no significant variation in this proportion by sex, age group, rural/urban residential status, employment and having ever been diagnosed with TB. Significantly more adults of mixed race (84.9 % [80.8–88.3]) and Indian (89.6 [82.0–94.2]) adults than black African adults (73.8 % [70.4–77.0]) knew that people with HIV are more likely to develop TB. Significantly more adults who had completed high school (80.6 % [75.8–84.6]) knewthat people with HIV are more likely to develop TB than those who did not complete high school (72.8 % [69.6–75.7]). Knowledge that people with HIV are more likely to develop TB was significantly higher among those receiving incomes above R 38 400 (approx. $3 200) per annum (84.7 % [79.7–88.6]) than those receiving no income (75.4 % [71.1–79.2]) and those receiving incomes of R 9 601-R 38400 (approx. $800–$3 200) per annum (69.9 % [65.5–73.9]). Significantly more adults who had initially learnt about TB from teachers (85.1 % [81.2–88.3]) knew that people with HIV are more likely to develop TB than those who did not learn about TB from teachers (73.3 % [70.0–76.4]).

### Composite TB knowledge score

The composite TB knowledge score (see Table 2), the sum of the six individual knowledge item scores, ranged from 0 to 6, with a mean score of 4.07 (SD = 1.28). Scores of 4–6 were achieved by 72.3 % of the respondents. Less than 2 % had a composite knowledge score of 0.

**Table 2:** Mean TB knowledge score by demographic characteristics, TB diagnosis and first source of information on TB

	Total knowledge score		
	Mean	95 % CI	n
Total	4.07	[3.99–4.14]	5945
Age			
18 to 24	4.07	[3.95–4.19]	1168
25 to 34	4.11	[4.01–4.22]	1405
35 to 44	4.09	[3.98–4.19]	1283
45 to 54	4.02	[3.89–4.15]	1218
55 to 64	3.98	[3.83–4.12]	871
Sex			
Female	4.10	[4.02–4.18]	3509
Male	4.03	[3.93–4.12]	2436
Race			
African	4.02	[3.93–4.10]	3824
White	4.22	[3.97–4.47]	312
Coloured	4.26	[4.16–4.36]	1394
Indian	4.15	[3.86–4.43]	415
Urban/Rural			
Rural	3.91	[3.79–4.02]	1850
Urban	4.14	[4.05–4.24]	4095
Completion of high school/equivalent			
Did not complete high school	3.91	[3.82–3.99]	3774
Completed high school (attained Gr12 or higher)	4.28	[4.17–4.39]	2171
Employment Status			
Unemployed	3.94	[3.85–4.03]	3183
Employed	4.19	[4.10–4.28]	2762
Annual income			
No income	4.00	[3.88–4.12]	2118
1–9600	4.05	[3.90–4.20]	682
9601–38400	3.91	[3.80–4.02]	1896
>–38401	4.35	[4.24–4.45]	1249
Have you ever been diagnosed with TB			
No	4.05	[3.98–4.13]	5487
Yes	4.27	[4.09–4.44]	458
Source of Knowledge: Where did you first learn about TB?			
Newspapers and magazines			
No	4.06	[3.98–4.14]	4568
Yes	4.08	[3.94–4.23]	1377
Radio			
No	3.97	[3.88–4.07]	3096
Yes	4.16	[4.06–4.27]	2849
Television			
No	3.92	[3.82–4.01]	3357
Yes	4.24	[4.15–4.34]	2588
Brochures, pamphlets or other printed materials			
No	4.03	[3.96–4.11]	5450
Yes	4.40	[4.20–4.60]	495
Health workers			
No	3.95	[3.86–4.05]	3710
Yes	4.25	[4.17–4.33]	2235
Family, friends, neighbours, colleagues			
No	4.09	[4.01–4.17]	4855
Yes	3.96	[3.80–4.13]	1090
Teachers			
No	3.99	[3.90–4.07]	4437
Yes	4.32	[4.21–4.44]	1508
Other sources of information			
No	4.08	[4.00–4.15]	5288
Yes	3.96	[3.78–4.14]	657

Mean TB composite knowledge did not differ significantly by sex, age group, and previous TB diagnosis. Mean knowledge was significantly higher among adults of mixed race (4.26 [4.16–4.36]) than black African (4.02 [3.93–4.10]) adults. Mean knowledge was significantly higher for those who had completed high school (4.28 [4.17–4.39]) than those who did not (3.91 [3.82–3.99]), for urban (4.14 [4.05–4.24]) residents than rural residents (3.91 [3.79–4.02]), and for the employed (4.19 [4.10–4.28] than the unemployed (3.94 [3.85–4.03]). Adults who received annual incomes of over R 38 400 (4.35 [4.24–4.45]) had higher mean knowledge than those who received no income (4.00 [3.88–4.12]), R <–9600 (4.05 [3.90–4.20]) and R 9 601–R38 400 per annum (3.91 [3.80–4.02]). Mean knowledge was significantly higher among those who were initially exposed to TB information from these channels/sources compared to those who were not: TV (4.24 [4.15–4.34] vs 3.92 [3.82–4.01]), brochures (4.40 [4.20–4.60] vs 4.03 [3.96–4.11]) health workers (4.25 [4.17–4.33] vs 3.95 [3.86–4.05]) and teachers (4.32 [4.21–4.44] vs 3.99 [3.90–4.07]).

### Determinants of composite TB knowledge score

Bivariate simple linear regression analyses (see Table 3) showed that the variables significantly associated with higher levels of the composite TB knowledge score in this survey were race (mixed race vs black African: Unstandardized co-efficient (β) = 0.246, *p* < 0.001) , residing in urban areas, completion of high school education, being employed, annual income (> R 38 400 vs no individual income: β = 0.346, *p* < 0.001), having ever been diagnosed with TB, and receiving information on TB from radio, television, brochures, health workers, and teachers. On including the variables that were found to be significant in the bivariate regression models, the multiple regression analyses (see Table 3) showed that race (mixed race vs black African: β =0.245, *p* < 0.001), being male (β = −0.101, *p* = 0.022), completion of high school (β =0.249, *p* < 0.001), employment (β =0.224, *p* = 0.005 ), having ever been diagnosed with TB (β =0.364, *p* < 0.001) and learning about TB from television (β =0.28, *p* < 0.001), brochures (β =0.261, *p* = 0.023), health workers (β =0.366, *p* < 0.001), and teachers (β =0.37, *p* < 0.001) were all significantly associated with higher levels of TB knowledge.

**Table 3:** Factors associated with composite TB knowledge score

Background characteristics	Composite TB Knowledge score					
	Simple regression			Multiple regression		
	Unstandardized co–eff (β)	s.e.	*p*-value	Unstandardized co-eff (β)	s.e.	*p*-value
Age (years)						
18 to 24						
25 to 34	0.044	0.071	0.539	−0.01	0.066	0.875
35 to 44	0.017	0.075	0.825	−0.055	0.077	0.475
45 to 54	−0.05	0.07	0.476	−0.084	0.072	0.245
55 to 64	−0.094	0.089	0.292	0.027	0.087	0.756
Sex: Male vs Female	−0.074	0.043	0.084	−0.101*	0.044	0.022
Race						
African	ref	-	-			
White	0.201	0.131	0.127	−0.021	0.125	0.867
Coloured	0.246***	0.064	<0.001	0.245***	0.067	<0.001
Indian	0.13	0.151	0.388	0.047	0.119	0.69
Urban vs Rural	0.236**	0.075	0.002	0.069	0.073	0.345
Completion of high school/equivalent						
Did not complete high school	ref	-	-			
Completed high school (attained Gr12 or higher)	0.369***	0.066	<0.001	0.249***	0.061	<0.001
Employed vs Unemployed	0.25***	0.053	<0.001	0.224**	0.079	0.005
Annual Income						
No income	ref	-	-			
<= R 9 600	0.051	0.085	0.545	0.004	0.086	0.961
R 9 601 - R 38 400	−0.087	0.078	0.265	−0.193	0.098	0.05
> = R 38 401	0.346***	0.076	<0.001	0.058	0.122	0.637
Ever diagnosed with TB	0.213*	0.094	0.023	0.364***	0.089	<0.001
Source of Knowledge: Where did you first learn about TB?						
Newspapers and magazines	0.021	0.082	0.803			
Radio	0.193**	0.066	0.004	0.14	0.072	0.052
Television	0.327***	0.061	<0.001	0.28***	0.066	<0.001
Brochures, pamphlets, other printed materials	0.367**	0.112	0.001	0.261*	0.115	0.023
Health Workers	0.299***	0.054	<0.001	0.366***	0.049	<0.001
Family, friends, neighbours, colleagues	−0.124	0.093	0.184			
Teachers	0.338***	0.069	<0.001	0.37***	0.072	<0.001
Other sources of information	−0.119	0.089	0.182			
(constant)				3.42***	0.089	<0.001

*p < 0.05, **p < 0.01, ***p < 0.001

F(20,423) = 11.11, *p* < 0.0001

R-squared = 0.0915

### Opinions on most effective sources of information on TB

Respondents were asked to select three sources of information they thought can most effectively reach people like themselves with information on TB, and were asked to choose the three most effective sources in their opinion. Radio (69.0 %) and television (64.8 %) were regarded as the most effective sources of information (Table 4), followed by health workers (43.3 %) and newspapers and magazines (39.2 %). Other sources that respondents chose were social media, campaigns, workshops and community programs.

**Table 4:** Sources of information that respondents thought would most effectively reach people like themselves with information on TB

	%	95 % CI	n
Radio	69	[66.5–71.4]	4048
Television	64.8	[61.9–67.5]	3844
Health workers	43.3	[40.5–46.2]	2643
Newspapers and magazines	39.2	[36.2–42.4]	2294
Brochures, posters and other printed materials	16.3	[14.7–18.1]	960
Teachers	15	[13.2–16.9]	983
Billboards	8.4	[7.2–9.8]	549
Family, friends, neighbours and colleagues	8.1	[6.9–9.5]	493
Religious leaders	7.7	[6.5–9.0]	514
Other	2.3	[1.6–3.3]	100

## Discussion

Overall, there was a relatively good level of TB knowledge in this study especially with respect to TB prevention, cure and TB and HIV co-existence. The low level of knowledge of TB symptoms is, however, of concern. Only about one fifth of the participants knew at least three out of six symptoms of TB. Given that TB is an easily transmittable disease it is important that the knowledge about the essential symptoms of TB, as defined by the WHO, is adequate at a population level. A lack of knowledge of the symptoms is likely to result in delayed health seeking, which in turn can result in on-going TB transmission and poorer health outcomes when individuals eventually access treatment [25]. The importance of the media in informing the public about the symptoms of TB was highlighted in this study. The results show that the knowledge about TB symptoms was significantly higher for those who first learnt about TB from newspapers and magazines than for those who did not. In addition, participants who had more knowledge about TB symptoms were exposed to TB knowledge through the radio, TV, brochures and health workers than those who were not.

The fact that at least two thirds of participants identified the correct transmission route of TB was an encouraging finding given that the disease is infectious. Importantly, at least four fifths of the sample correctly identified the behaviour to be enacted to prevent the transmission of TB. Knowledge of TB prevention methods was higher among those who were employed, earning a higher salary and having the first exposure to TB knowledge through the radio, TV, brochures, and health workers. These findings lend support to the existing literature regarding the fact that a better socio-economic status is associated with increased levels of health literacy [26, 27]. Moreover, the role of the media and health workers are also important sources of knowledge about TB prevention methods.

Over four fifths of the sample knew that TB is curable. They also knew the drug treatment regimen required for the treatment of the disease. Significantly more participants of mixed race than black African participants, those who completed high school than those who did not, those who had TB in their life-time than those who did not, and those who were initially exposed to TB knowledge from their health workers than those who did not had significantly more knowledge about the curability and drug treatment necessary to cure TB. It is clear that there are a number of factors, including race, education, personal experience and the influence of public health care practitioners, that influence the level of knowledge about the curability of TB and the specific drug treatment required for this. Given the high prevalence of TB in SA, one may assume that most communities would have had personal exposure to the disease either as someone who has been infected in their life-time, or as someone who has been exposed to and affected by TB through a family member, community member , or work colleague who might have been diagnosed with the disease.

More than four fifths of the sample knew that individuals who have TB should be tested for HIV, and at least three quarters knew that individuals with HIV are more likely to develop TB. Significantly more participants of mixed race than black Africans, more educated than not educated and those who were employed than those who were not knew that individuals with TB should be tested for HIV. Race (mixed race and Indian compared to black African), higher levels of education and income, and health messaging provided by educators were significant factors associated with the knowledge of TB and HIV co-existence. The awareness of this co-existence between TB and HIV is encouraging given the burden of disease status that both TB and HIV have in SA [8].

At least two thirds of the sample achieved a mean composite TB knowledge score (4.07). Significantly more participants of mixed race than black African participants, those who completed high school than those who did not and those who were employed compared to those who were not, those who had a higher income than those with a lower income or no income, those who were diagnosed with TB in their life-time than those who were not and those who were initially exposed to information about TB through the brochures, health workers and teachers than those who were not, had a higher mean composite knowledge score for TB. Many factors such as race (mixed race vs black African), employment, income, personal experience with TB disease, the media and public service agents (health workers and teachers) are associated with a relatively good mean composite knowledge score. This finding demonstrates the complexity in addressing the acquirement of TB knowledge and points to the fact that any intervention aimed at improving TB knowledge at a population level needs to factor in this complexity.

In the final analysis the factors that were the strongest predictors of higher levels of TB knowledge were race (mixed race vs black African), gender (being male), education (completion of high school), being employed, having been diagnosed with TB in a life-time, and learning about TB through the media (TV) and public service staff (namely, health workers and teachers). The fact that being of mixed race compared to being black African is a predictor of higher levels of TB knowledge is perhaps due to the fact that individuals of mixed race have been historically affected by TB and continue to have high prevalence rates of the disease in the country [8]. Of particular value in the study findings is that we were able to ascertain, on a national scale, the importance of TB knowledge dissemination through the media, and health and educational agents. These results can be used to inform the media, as an agent of change, about their role in reducing TB disease burden in SA.

## Conclusion

The strength of this study is that SANHANES I is the first to produce nationally representative data to help draw conclusions about the predictors of TB knowledge. Perhaps a major limitation is that when questions were asked about the initial learning about TB, multiple responses were allowed. It is possible that participants had recall bias and were not accurately reporting the first set of knowledge exposure through the media or other sources. Another limitation of the study is that participants possibly experienced “respondent fatigue” after answering the multiple questions that were asked. It is also possible that a proportion of the participants did not fully understand the questions.

Despite these limitations, the results of this study highlight ways to improve more accurate knowledge about TB at a population level in SA. The results show clearly how media campaigns and knowledge transmission can be implemented and directed at certain target groups to encourage detection, treatment and health care seeking behaviour for TB. Media campaigns should be tailored for both rural and urban communities.

Given the fact that HIV counselling and testing (HCT) in SA now includes education about TB co-morbidity, and the fact that there has recently been an increase in media coverage about TB, it is expected that there will be more accurate knowledge dissemination about TB disease. Media programs have the potential to disseminate accurate information about the disease (e.g. symptoms and treatment) as well as promote treatment-seeking behaviour and adherence to treatment [28]. In Pakistan, for example, Turk et al. [29] evaluated the Advocacy, Communication and Social Mobilization (ACSM) national TB campaign across 57 districts. The researchers found that there were significant differences in knowledge, attitudes, and intended behaviors towards TB between groups who were unaware of media and community based activities and groups who were aware. Broadcasting media appears to be closely linked to positive outcomes. In another study, listening to the radio was found to be associated with correct knowledge of the transmission of the disease [1]. In Bangladesh, advertisements that raise awareness about TB and TB treatment were identified as influencing health-seeking practices [30].

The results of the study also highlight the fact that better socio-economic status, i.e. higher levels of education and income are associated with better TB knowledge. Given this finding, government agencies and policy makers across sectors need to improve their strategies to combat TB by encouraging economic and social development. The private sector also has a role to play in addressing structural barriers [31, 32] either on their own or through private-public partnerships. In a country, such as SA, that has a high index of inequality with a Gini-co-efficient of 0.66 [33] a more concerted effort has to be made to provide educational and employment opportunities to underserved communities.

## Additional files

Additional file 1: Visiting Point Questionnaire. (PDF 586 kb) Additional file 2: Adult Questionnaire: 15 years and older. (PDF 294 kb) Additional file 3: Correct knowledge for the six individual knowledge items by demographic characteristics, TB diagnosis and sources of information on TB. (DOCX 33 kb)
