# Supplementary material for: Predictors of knowledge about tuberculosis: results from SANHANES I, a national, cross-sectional household survey in South Africa
Source: BMC Public Health. 2016 Mar 18;16:276. doi: 10.1186/s12889-016-2951-y (PMC4797251; doi:10.1186/s12889-016-2951-y)
Supplement: Additional file 1: — Visiting Point Questionnaire. (PDF 586 kb) [file 12889_2016_2951_MOESM1_ESM.pdf]

Visiting Point Questionnaire Number: ______________________________

THE SOUTH AFRICAN NATIONAL HEALTH AND NUTRITION EXAMINATION SURVEY, 2011/2012

(SANHANES-1)

Visiting Point Questionnaire

A. Geographic Particulars

Province								
Enumerator area (EA)								
Visiting point (VP) number (taken from map)								
Visiting point (VP) address					C	O	D	E

B. Number of Households at Visiting Point

Draw a diagram of the visiting point / stand. If more than one household is present, indicate how many separate households are resident at this visiting point. Allocate a number to each household and use the Kish grid to select only one household

Number of households at visiting point

The assigned 'number' of the selected household

C. Interview Details

	Year	Month		Day		Time code	Response code
First visit	2012						
Second visit	2012						
Third visit	2012						
Fourth visit	2012						
Fifth visit	2012						
						Final response code	

Time code
1 = Morning till 12h00
2 = 12h01-15h00
3 = 15h01-18h00
4 = 18h01-21h00
5 = 21h01 and later

Response code
1 = Interview completed
2 = Interview partly completed and another appointment made
3 = Appointment made for interview
4 = Not a valid visiting point
5 = No one living here (unoccupied)
6 = No one at home
7 = No one at home for duration of the survey
8 = Refusal by household head
9 = No one to consent (specify)……………………………
10 = Incapacitated (specify)…………………………………..
11 = Other (Specify) …………………………………………..

Fieldworker	Name						
	Staff number						
Interview starting time:			H	H	:	M	M

1 INSTRUCTION TO INTERVIEWER	List the persons who usually live in your household (sleep in your household for at least four nights a week) starting with the head of household.

SECTION A HOUSEHOLD MEMBERS AND THEIR CHARACTERISTICS

PERSON NO.	PERSON NAME	AGE	SEX	RELATIONSHIP	MARITAL STATUS	SPOUSE / PARTNER	RACE	INDIVIDUAL QUESTIONNAIRE NUMBER
		How old is (name)?<br>Write 00 if under 1 year	Is (name) male or female?	What is (name's) relationship to the head of the household?	What is (name's) PRESENT marital status?<br>If code 03-06 - go to q8	Who in this household is (name's) spouse or partner?<br>Write the person no. of the spouse / partner. Write 98 if he/she does not live in the h/hold	How would (name) describe him / herself in terms of race	**FOR FIELDWORKER USE ONLY**<br>Complete once all individual interviews are done
**(1)**	**(2)**	**(3)**	**(4)**	**(5)**	**(6)**	**(7)**	**(8)**	**(9)**
		In years	M F					
1			1 2					
2			1 2					
3			1 2					
4			1 2					
5			1 2					
6			1 2					
7			1 2					
8			1 2					
9			1 2					
10			1 2					
				01 = Head/Acting head<br>02 = Wife/husband/ partner<br>03 = Son/daughter<br>04 = Son/daughter in law<br>05 = Grandchild<br>06 = Mother/father<br>07 = Mother/father in law<br>08 = Brother/sister<br>09 = Niece/nephew<br>10 = Other relative<br>11 = Adopted/foster/ stepchild<br>12 = Not related<br>13 = Don't know	1 = Married<br>2 = Living together<br>3 = Never married<br>4 = Widowed<br>5 = Separated<br>6 = Divorced<br>7 = Civil Union		1 = African<br>2 = White<br>3 = Coloured<br>4 = Indian/Asian<br>5 = Other	

SECTION A HOUSEHOLD MEMBERS AND THEIR CHARACTERISTICS

PERSON NO.	LANGUAGE	SCHOOL ATTENDANCE	LEVEL OF EDUCATION	PRESENT EMPLOYMENT
	Language spoken most often at home	Does (name) currently attend an educational institution? Attendance includes all part time and full time studies, whether in person or as a distance learner	What is the highest level of education that (name) has completed)?	How would you describe (name's) present employment situation?
**(1)**	**(10)**	**(11)**	**(12)**	**(13)**
		(Only ages 5-24)	(Only persons aged 7 and older)	(Only persons aged 18- 64)
1				
2				
3				
4				
5				
6				
7				
8				
9				
10				
	01 = Afrikaans<br>02 = English<br>03 = Isindebele<br>04 = Isiswati<br>05 = Isixhosa<br>06 = Isizulu<br>07 = Sesotho<br>08 = Sepedi<br>09 = Setswana<br>10 = Tshivenda<br>11 = Xitsonga<br>12 = Other African<br>13 = Other European/ Asian	1 = Yes<br>2 = No<br>3 = Don't know	98 = No schooling<br>00 = Grade R/Pre-school<br>01 = Grade 1/ Sub A<br>02 = Grade 2/ Sub B<br>03 = Grade 3/ STD 1/ ABET 1<br>04 = Grade 4/ STD 2<br>05 = Grade 5/ STD 3/ ABET 2<br>06 = Grade 6/ STD 4<br>07 = Grade 7/ STD 5/ ABET 3<br>08 = Grade 8/ STD 6/ Form1<br>09 = Grade 9/ STD 7/ABET 4/Form 2<br>10 = Grade 10/ STD 8/ Form 3<br>11 = Grade 11/ STD 9/ Form 4<br>12 = Grade 12/ STD 10/ Form 5<br>13 = NTC Level 2 - 4<br>14 = NTC 4/ N4 - NTC 6/ N6<br>15 = Certificate/Diploma with less than Grade 12/ STD 10<br>16 = Certificate / Diploma with Grade 12/ STD 10<br>17 = Higher Degrees<br>18 = Other	01 = Housewife, homemaker, not looking for work<br>02 = Housewife, homemaker, looking for work<br>03 = Unemployed, looking for work<br>04 = Unemployed, not looking for work<br>05 = Work in informal sector, not looking for permanent work<br>06 = Sick/ disabled and unable to work<br>07 = Student/pupil/learner<br>08 = Self employed – Full time (40 hrs or more per week)<br>09 = Self-employed - Part time (less than 40 hrs per week)<br>10 = Employed - Part time (less than 40 hrs per week)<br>11 = Employed – Full time (40 hrs or more per week)<br>12 = Employed (Seasonal Work)<br>13 = Retired<br>14 = Other

SECTION A	Continuation if more than 10 people in the household							
PERSON NO.	PERSON NAME	AGE	SEX	RELATIONSHIP	MARITAL STATUS	SPOUSE / PARTNER	RACE	INDIVIDUAL QUESTIONNAIRE NUMBER
		How old is (name)?<br>Write 00 if under 1 year	Is (name) male or female?	What is (name's) relationship to the head of the household?	What is (name's) PRESENT marital status?<br>If code 03-06 - go to q8	Who in this household is (name's) spouse or partner?<br>Write the person no. of the spouse / partner. Write 98 if he/she does not live in the h/hold	How would (name) describe him / herself in terms of race	**FOR FIELDWORKER USE ONLY**<br>Complete once all individual interviews are done
**(1)**	**(2)**	**(3)**	**(4)**	**(5)**	**(6)**	**(7)**	**(8)**	**(9)**
		In years	M F					
11			1 2					
12			1 2					
13			1 2					
14			1 2					
15			1 2					
16			1 2					
17			1 2					
18			1 2					
19			1 2					
20			1 2					
				01 = Head/Acting head<br>02 = Wife/husband/ partner<br>03 = Son/daughter<br>04 = Son/daughter in law<br>05 = Grandchild<br>06 = Mother/father<br>07 = Mother/father in law<br>08 = Brother/sister<br>09 = Niece/nephew<br>10 = Other relative<br>11 = Adopted/foster/ stepchild<br>12 = Not related<br>13 = Don't know	1 = Married<br>2 = Living together<br>3 = Never married<br>4 = Widowed<br>5 = Separated<br>6 = Divorced<br>7 = Civil Union		1 = African<br>2 = White<br>3 = Coloured<br>4 = Indian/Asian<br>5 = Other	

SECTION A — Continuation if more than 10 people in the household

PERSON NO.	LANGUAGE	SCHOOL ATTENDANCE	LEVEL OF EDUCATION		PRESENT EMPLOYMENT
	Language spoken most often at home	Does (name) currently attend an educational institution? Attendance includes all part time and full time studies, whether in person or as a distance learner	What is the highest level of education that (name) has completed)?		How would you describe (name's) present employment situation?
**(1)**	**(10)**	**(11)**	**(12)**		**(13)**
		(Only ages 5-24)	(Only persons aged 7 and older)		(Only persons aged 18- 64)
11					
12					
13					
14					
15					
16					
17					
18					
19					
20					
	01 = Afrikaans<br>02 = English<br>03 = Isindebele<br>04 = Isiswati<br>05 = Isixhosa<br>06 = Isizulu<br>07 = Sesotho<br>08 = Sepedi<br>09 = Setswana<br>10 = Tshivenda<br>11 = Xitsonga<br>12 = Other African<br>13 = Other European/ Asian	1 = Yes<br>2 = No<br>3 = Don't know	98 = No schooling<br>00 = Grade R/Pre-school<br>01 = Grade 1/ Sub A<br>02 = Grade 2/ Sub B<br>03 = Grade 3/ STD 1/ ABET 1<br>04 = Grade 4/ STD 2<br>05 = Grade 5/ STD 3/ ABET 2<br>06 = Grade 6/ STD 4<br>07 = Grade 7/ STD 5/ ABET 3<br>08 = Grade 8/ STD 6/ Form1	09 = Grade 9/ STD 7/ABET 4/Form 2<br>10 = Grade 10/ STD 8/ Form 3<br>11 = Grade 11/ STD 9/ Form 4<br>12 = Grade 12/ STD 10/ Form 5<br>13 = NTC Level 2 - 4<br>14 = NTC 4/ N4 - NTC 6/ N6<br>15 = Certificate/Diploma with less than Grade 12/ STD 10<br>16 = Certificate / Diploma with Grade 12/ STD 10<br>17 = Higher Degrees<br>18 = Other	01 = Housewife, homemaker, not looking for work<br>02 = Housewife, homemaker, looking for work<br>03 = Unemployed, looking for work<br>04 = Unemployed, not looking for work<br>05 = Work in informal sector, not looking for permanent work<br>06 = Sick/ disabled and unable to work<br>07 = Student/pupil/learner<br>08 = Self employed – Full time (40 hrs or more per week)<br>09 = Self-employed - Part time (less than 40 hrs per week)<br>10 = Employed - Part time (less than 40 hrs per week)<br>11 = Employed – Full time (40 hrs or more per week)<br>12 = Employed (Seasonal Work)<br>13 = Retired<br>14 = Other

SECTION A	HOUSEHOLD MEMBERS AND THEIR CHARACTERISTICS		
PERSON NO.	OCCUPATION	SOURCE OF INCOME	INCOME CATEGORY
	What kind of work does (name) usually do in his/her main job/business<br><br>Write a short description of the occupation	What is (name's) main source of income?	What is the income category that best describes (name's) gross monthly / annual income before deductions and including all sources of income?
**(1)**	**(14)**	**(15)**	**(16)**
	(Only persons aged 18- 64)	(Only persons aged 18 years and older)	(Only persons aged 18 years and older)
1			
2			
3			
4			
5			
6			
7			
8			
9			
10			
		1 = Salaries and / or wages<br>2 = Remittances<br>3 = Pension/ Grants / UIF<br>4 = Sale of products and services<br>5 = No income<br>6 = Refused to answer<br>7 = Don't know	Monthly<br>01 = no income<br>02 = R1 – R400<br>03 = R401 – R800<br>04 = R801 – R1 600<br>05 = R1 601 – R3 200<br>06 = R3 201 – R6 400<br>07 = R6 401 – R12 800<br>08 = R12 801 – R25 600<br>09 = R25 601 – R51 200<br>10 = R51201 – R102 400<br>11 = R102 401 – R204 800<br>12 = R 204 801 or more<br>13 = Refused to answer<br><br>Annual<br>01 = no income<br>02 = R1 – R4 800<br>03 = R4 801 – R9 600<br>04 = R9 601 – R19 200<br>05 = R19 201 – R38 400<br>06 = R38 401 – R76 800<br>07 = R76 801 – R153 600<br>08 = R153 601 – R307 200<br>09 = R307 201 – R614 400<br>10 = R614 401 – R1 228 800<br>11 = R1 228 801 – R2 457 600<br>12 = R 457 601 or more<br>13 = Refused to answer

2 INSTRUCTION TO INTERVIEWER	Just to make sure that I have a complete listing: I listed _____ persons.<br>Are there any other persons such as small children or infants that we have not listed? Are there any other people who may not be members of your family, such as domestic servants, lodgers or friends who usually sleep here for at least four nights a week?<br>LIST ANY ADDITIONAL MEMBERS IN THE HOUSEHOLD SCHEDULE

WRITE THE "PERSON NUMBER" OF THE MAIN RESPONDENT WHO PROVIDED THE INFORMATION		
DID THE HOUSEHOLD HEAD CONSENT TO FOLLOW UP IN THE FOLLOW UP STUDY	Y	N

**SECTION A**	**Continuation if more than 10 people in the household**		
PERSON NO.	OCCUPATION	SOURCE OF INCOME	INCOME CATEGORY
	What kind of work does (name) usually do in his/her main job/business<br>Write a short description of the occupation	What is (name's) main source of income?	What is the income category that best describes (name's) gross monthly / annual income before deductions and including all sources of income?
**(1)**	**(14)**	**(15)**	**(16)**
	(Only persons aged 18- 64)	(Only persons aged 18 years and older)	(Only persons aged 18 years and older)
11		☐	☐☐
12		☐	☐☐
13		☐	☐☐
14		☐	☐☐
15		☐	☐☐
16		☐	☐☐
17		☐	☐☐
18		☐	☐☐
19		☐	☐☐
20		☐	☐☐
		1 = Salaries and / or wages<br>2 = Remittances<br>3 = Pension/ Grants / UIF<br>4 = Sale of products and services<br>5 = No income<br>6 = Refused to answer<br>7 = Don't know	Monthly<br>01 = no income<br>02 = R1 – R400<br>03 = R401 – R800<br>04 = R801 – R1 600<br>05 = R1 601 – R3 200<br>06 = R3 201 – R6 400<br>07 = R6 401 – R12 800<br>08 = R12 801 – R25 600<br>09 = R25 601 – R51 200<br>10 = R51201 – R102 400<br>11 = R102 401 – R204 800<br>12 = R 204 801 or more<br>13 = Refused to answer<br><br>Annual<br>01 = no income<br>02 = R1 – R4 800<br>03 = R4 801 – R9 600<br>04 = R9 601 – R19 200<br>05 = R19 201 – R38 400<br>06 = R38 401 – R76 800<br>07 = R76 801 – R153 600<br>08 = R153 601 – R307 200<br>09 = R307 201 – R614 400<br>10 = R614 401 – R1 228 800<br>11 = R1 228 801 – R2 457 600<br>12 = R 457 601 or more<br>13 = Refused to answer

2 INSTRUCTION TO INTERVIEWER	Just to make sure that I have a complete listing: I listed _____ persons.<br>Are there any other persons such as small children or infants that we have not listed? Are there any other people who may not be members of your family, such as domestic servants, lodgers or friends who usually sleep here for at least four nights a week? LIST ANY ADDITIONAL MEMBERS IN THE HOUSEHOLD SCHEDULE

WRITE THE "PERSON NUMBER" OF THE MAIN RESPONDENT WHO PROVIDED THE INFORMATION		
DID THE HOUSEHOLD HEAD CONSENT TO FOLLOW UP IN THE FOLLOW UP STUDY	Y	N

[3] INSTRUCTION TO INTERVIEWER	Record the answers by circling a response or by writing the answer in the space provided.<br>Please note that <u>only one response is allowed per question</u> unless another instruction is given.<br>Please note that <u>coding categories should NOT be read to the participant</u> unless another instruction is given

SECTION B-1 FOOD SECURITY

NO.	QUESTIONS AND FILTERS	CODING CATEGORIES	SKIP
	**Access and availability to food has become very topical in South Africa.**<br>**The next questions measure access and availability of food in households across the country.**		
1	Who is mainly responsible for food preparation?	Name: ....<br>Person No from the household schedule ………… ☐☐	
2	Who decides on what types of food are bought for the household?	Name: ....<br>Person No from the household schedule ………… ☐☐	
3	Who is mainly responsible for feeding / serving the child/children?<br>Multiple responses possible<br>If the person responsible is not listed in the household schedule write '00'<br>If there are no children in the household write '88'	1) Name: ....<br>1) Person No from the household schedule ………… ☐☐<br>2) Name: ....<br>2) Person No from the household schedule ………… ☐☐	
4	Who decides how much money is spent on food?<br>Multiple responses possible	1) Name: ....<br>1) Person No from the household schedule ………… ☐☐<br>2) Name: ....<br>2) Person No from the household schedule ………… ☐☐	
5	How much money is spent on food weekly?	R 0 – R 50 .... 1<br>R 50 – R 100 .... 2<br>R 100 – R 150 .... 3<br>R 150 – R 200 .... 4<br>R 200 – R 250 .... 5<br>R 250 – R 300 .... 6<br>R 300 – R 350 .... 7<br>R 350 – R 400 .... 8<br>More than R400 .... 9<br>Don't know 10	

SECTION B – 2	HUNGER SCALE		
NO.	QUESTIONS AND FILTERS	CODING CATEGORIES	SKIP
6a	Does your household ever run out of money to buy food?	Yes .... 1<br>No 2	 ➢7a
6b	Has it happened in the past 30 days?	Yes .... 1<br>No 2	 ➢7a
6c	Has it happened 5 or more days in the past 30 days?	Yes .... 1<br>No 2	
7a	Do you ever rely on a limited number of foods to feed your children because you are running out of money to buy food for a meal?	Yes .... 1<br>No 2	 ➢8a
7b	Has it happened in the past 30 days?	Yes .... 1<br>No 2	 ➢8a
7c	Has it happened 5 or more days in the past 30 days?	Yes .... 1<br>No 2	
8a	Do you ever cut the size of meals or skip meals because there is not enough money for food?	Yes .... 1<br>No 2	 ➢9a
8b	Has it happened in the past 30 days?	Yes .... 1<br>No 2	 ➢9a
8c	Has it happened 5 or more days in the past 30 days?	Yes .... 1<br>No 2	
9a	Do you ever eat less than you should because there is not enough money for food?	Yes .... 1<br>No 2	 ➢10a
9b	Has it happened in the past 30 days?	Yes .... 1<br>No 2	 ➢10a
9c	Has it happened 5 or more days in the past 30 days?	Yes .... 1<br>No 2	
10a	Do your children ever eat less than you feel they should because there is not enough money for food?	Yes .... 1<br>No 2	 ➢11a
10b	Has it happened in the past 30 days?	Yes .... 1<br>No 2	 ➢11a
10c	Has it happened 5 or more days in the past 30 days?	Yes .... 1<br>No 2	
11a	Do your children ever say they are hungry because there is not enough food in the house?	Yes .... 1<br>No 2	 ➢12a
11b	Has it happened in the past 30 days?	Yes .... 1<br>No 2	 ➢12a
11c	Has it happened 5 or more days in the past 30 days?	Yes .... 1<br>No 2	
12a	Do you ever cut the size of your children's meals or do they ever skip meals because there is not enough money to buy food?	Yes .... 1<br>No 2	 ➢13a
12b	Has it happened in the past 30 days?	Yes .... 1<br>No 2	 ➢13a
12c	Has it happened 5 or more days in the past 30 days?	Yes .... 1<br>No 2	
13a	Do any of your children go to bed hungry because there is not enough money to buy food?	Yes .... 1<br>No 2	 ➢14a
13b	Has it happened in the past 30 days?	Yes .... 1<br>No 2	 ➢14a
13c	Has it happened 5 or more days in the past 30 days?	Yes .... 1<br>No 2	

SECTION B-3 — MONTHS OF FOOD SHORTAGES

NO.	QUESTIONS AND FILTERS	CODING CATEGORIES	SKIP
14a	In the <u>past 12 months</u>, were there times when members of your household went hungry because there was not enough food in the house to eat?	Yes....1<br>No....2	 ➢15a
14b	Which were the months (in the last 12 months) in which you experienced a lack of food or money such that one or more members of your household had to go hungry?<br><br>Multiple responses possible	January....1<br>February....2<br>March....3<br>April....4<br>May....5<br>June. ....6<br>July....7<br>August....8<br>September....9<br>October....10<br>November....11<br>December....12	
15a	In the <u>past 12 months</u>, were there times that there was a shortage of money in the household?	Yes....1<br>No....2	 ➢C1
15b	Which were the months (in the last 12 months) in which there was a shortage of money?<br><br>Multiple responses possible	January....1<br>February....2<br>March....3<br>April....4<br>May....5<br>June. ....6<br>July....7<br>August....8<br>September....9<br>October....10<br>November....11<br>December....12	

SECTION C — ALCOHOL

NO.	QUESTIONS AND FILTERS	CODING CATEGORIES	SKIP
	**The following questions are about your feelings on laws concerning alcohol**		
1	At present the legal drinking age is 18 years. Do you think that the legal drinking age should be………..<br><br>Read options	Increased (raised)....1<br>Decreased or lowered....2<br>Remain the same....3<br>Don't know (no opinion)....4	
2	Do you think that taxes on alcoholic drinks should be………….<br><br>Read options	Increased (raised)....1<br>Decreased or lowered....2<br>Remain the same....3<br>Don't know (no opinion)....4	
3	Do you think that alcoholic drinks should have warning labels about possible health hazards?	Yes....1<br>No....2<br>Don't know (no opinion)....3	

SECTION C	ALCOHOL		
NO.	QUESTIONS AND FILTERS	CODING CATEGORIES	SKIP
	**The following questions are on alcohol use in your household and neighbourhood**		
4	Who in your household consumes alcohol?<br><br>Multiple responses possible	Everyone .... 1<br>Adult Men .... 2<br>Adult Women .... 3<br>Teenage boys .... 4<br>Teenage girls .... 5<br>Nobody .... 6<br>Don't know 7	 <br><br><br><br>➢ 8<br>➢ 8
5	How serious is the problem of drinking and driving amongst members of your household?	Very serious .... 1<br>Serious .... 2<br>Not very serious .... 3<br>Not a problem .... 4<br>Don't know (no opinion) 5	
6	How serious are the problems of misuse of alcohol in your household?<br>Probe<br>Has any member of the family failed to fulfil obligations at work, school or home as a result of alcohol use eg. repeated absences or poor work performance, suspensions or expulsions from school, neglect of children or household	Very serious .... 1<br>Serious .... 2<br>Not very serious .... 3<br>Not a problem .... 4<br>Don't know (no opinion) 5	
7	How serious is the problem of violence or disturbances due to alcohol use in your home?	Very serious .... 1<br>Serious .... 2<br>Not very serious .... 3<br>Not a problem .... 4<br>Don't know (no opinion) 5	
8	How serious is the problem of drinking and driving in your neighbourhood?	Very serious .... 1<br>Serious .... 2<br>Not very serious .... 3<br>Not a problem .... 4<br>Don't know (no opinion) 5	
9	How serious is the problem of public fights or disturbances due to alcohol use in your neighbourhood?	Very serious .... 1<br>Serious .... 2<br>Not very serious .... 3<br>Not a problem .... 4<br>Don't know (no opinion) 5	
	**If no one in the household consumes alcohol, skip to Section D**		➢Section D
10	Do you or members of the household snack while drinking alcohol?	Yes .... 1<br>No .... 2<br>Don't know 3	
11	Do you or members of the household eat before you drink?	Yes .... 1<br>No .... 2<br>Don't know 3	

SECTION D	HEALTH INSURANCE - ASK HOUSEHOLD HEAD		
NO.	QUESTIONS AND FILTERS	CODING CATEGORIES	SKIP
1	Where do you usually get your health care from?	Private doctor's office....1<br>Private clinic or health care facility....2<br>Private hospital....3<br>Public clinic or health care facility....4<br>Public hospital....5<br>Charity or church run clinic....6<br>Charity or church run hospital....7<br>Home visit....8<br>Other (Specify)....9	
2	Do you have private medical aid / health insurance either in your own name or through another family member?	Yes, in own name....1<br>Yes, through a family member....2<br>No....3	➢ 4<br>➢ 4
3	If you are not covered by a medical scheme, which of the following do you do?<br>Read options	Pay out of your pocket to see a doctor or buy medicines from pharmacies....1<br>Rely on public sector for all health care services....2<br>Don't know....3	➢ 6<br>➢ 6<br>➢ 6
4	Does your medical aid / health insurance cover any part of the cost of prescription medication?	Yes....1<br>No....2<br>Don't know....3	
5	Overall, how satisfied are you with your medical aid / health insurance coverage?	Very satisfied....1<br>Satisfied....2<br>Neither satisfied nor dissatisfied....3<br>Dissatisfied....4<br>Very dissatisfied....5<br>Don't know....6	
6	How satisfied or dissatisfied are you with the quality of the health care you receive?	Very satisfied....1<br>Satisfied....2<br>Neither satisfied nor dissatisfied....3<br>Dissatisfied....4<br>Very dissatisfied....5<br>Don't know....6	
7	How satisfied or dissatisfied are you with the cost of your health care?	Very satisfied....1<br>Satisfied....2<br>Neither satisfied nor dissatisfied....3<br>Dissatisfied....4<br>Very dissatisfied....5<br>Don't know....6	
8	In the past 12 months, have you had difficulty affording the cost of necessary medical care?	Yes....1<br>No....2<br>Don't know....3	
9	In the past 12 months have you had difficulty affording the cost of prescription medication?	Yes....1<br>No....2<br>Don't know....3	
10	In the past 12 months, have you put off or postponed getting the health care you needed?	Yes....1<br>No....2<br>Don't know....3	

SECTION D HEALTH INSURANCE - ASK HOUSEHOLD HEAD

NO.	QUESTIONS AND FILTERS	CODING CATEGORIES	SKIP
11	Please tell me how serious a problem you think it is that many South Africans lack access to a medical aid scheme and private health care facilities.<br><br>Read options	Very serious problem ....1<br>Fairly serious problem ....2<br>Somewhat serious problem ....3<br>Not a serious problem ....4<br>Don't know 5	
12	Is it right or wrong that people with higher incomes can afford better health care than people with lower incomes?<br><br>Read options	Definitely right ....1<br>Somewhat right ....2<br>Neither right nor wrong ....3<br>Somewhat wrong ....4<br>Definitely wrong ....5<br>Can't choose 6	
13	In the past <u>6 months</u>, have you seen, read or heard any news or information about a proposal by government to introduce a programme to provide national health insurance for all South Africans?	Yes ....1<br>No ....2<br>Don't know 3	 ➢ 15<br>➢ 15
14	Would you say that you know a lot, a fair amount, a little or not yet enough about the proposed national health insurance?	A lot ....1<br>A fair amount ....2<br>A little ....3<br>Not yet enough 4	
	**We are now going to talk about some of the changes government is planning with regard to health care in South Africa. The government wants to create a National Health Insurance, which is a system in which everyone is covered by health insurance and people contribute according to ability to pay and use health services according to their needs.**		
15	Should national health insurance for all South Africans be ……………………………..?<br><br>Read options	Top priority ....1<br>Important but lower priority ....2<br>Not too important ....3<br>Should not be done ....4<br>Don't know 5	
16	Which priority do you think the country should focus on first …………………………………...?<br><br>Read options	Make health care better and more affordable ....1<br>Provide health insurance for all South Africans ....2<br>Don't know 3	
17	Do you think that as a country we could afford to provide everyone with all the health and medical services which they need, <u>OR</u> would that cost more than the country can afford?	We could afford to ....1<br>Would cost too much ....2<br>Don't know 3	
18	Which of the following do you think is more important………………………………….?<br><br>Read options	Health Insurance for all, even if it means raising taxes ....1<br>Holding down taxes, even if it means some people don't have health insurance ....2<br>Don't know 3	
19	Which of the following would you prefer……………?<br><br>Read options	Current medical aid system - where some people and their families get their medical aid through employers, while many people have no medical aid ....1<br>Universal national health insurance program - where everyone is covered under a program financed by taxpayers ....2<br>Don't know 3	

SECTION D	HEALTH INSURANCE - ASK HOUSEHOLD HEAD		
NO.	QUESTIONS AND FILTERS	CODING CATEGORIES	SKIP
20	If the planned national health insurance lowered your health care costs and provided cover to all South Africans, but limited your choice of doctor, hospital or treatment, would you support or oppose such a plan?	Support....1<br>Oppose....2<br>Don't know....3	
21	If there was a health insurance scheme that would cover the health care costs of all South Africans, whom would you trust most to run this scheme?<br><br>Read options	Organisation linked to government....1<br>A private organisation....2<br>Don't know....3	
22	To what extent do you agree or disagree that it would be cheaper to have a government operated national health insurance compared to the current arrangements?	Strongly agree....1<br>Agree....2<br>Neither agree nor disagree....3<br>Disagree....4<br>Strongly disagree....5<br>Don't know....6	
23	If government goes ahead and introduces the health insurance scheme that would cover the health care costs for all South Africans, do you think you and your family would be financially…………………..?<br><br>Read options	Better off....1<br>Worse off....2<br>It would not have much effect....3<br>Don't know....4	
24	If government goes ahead and introduces the health insurance scheme that would cover the health care costs for all South Africans, do you think the country as a whole would be overall …………………….?<br><br>Read options	Better off....1<br>Worse off....2<br>It would not have much effect....3<br>Don't know....4	
25	Do you believe that the proposed national health insurance would make the quality of the health care you receive better, worse, or about the same as now?	Better....1<br>Worse....2<br>About the same....3<br>Don't know....4	

SECTION E	HOUSING, HOUSEHOLD GOODS AND SERVICES		
NO.	QUESTIONS AND FILTERS	CODING CATEGORIES	SKIP
1	Type of dwelling?<br><br>(FIELDWORKER: Please observe then record your observation)	House of brick/ concrete block structure on a separate stand or yard or on a farm....1<br>Traditional dwelling / hut / structure made of traditional materials....2<br>Flat or apartment in a block of flats....3<br>Cluster house in complex....4<br>Townhouse (semi-detached house in complex)....5<br>Semi-detached house....6<br>House / flat / room in backyard....7<br>Informal dwelling / shack in back yard....8<br>Informal dwelling / shack not in back yard....9<br>Room or flatlet on a property or larger dwelling / servant's quarters / granny flat....10<br>Caravan / tent....11<br>Other (Specify)....12	

SECTION E	HOUSING, HOUSEHOLD GOODS AND SERVICES		
NO.	QUESTIONS AND FILTERS	CODING CATEGORIES	SKIP
2	Construction material of the walls and roof?<br><br>(FIELDWORKER: Please observe then record your observation)<br><br>Multiple responses possible	Brick....1<br>Cement block / concrete....2<br>Corrugated iron / zinc....3<br>Wood....4<br>Plastic....5<br>Cardboard....6<br>Mud and cement mix....7<br>Wattle and daub....8<br>Tile....9<br>Mud....10<br>Thatch / grass....11<br>Asbestos....12<br>Other (Specify)....13	
3	How many rooms does your dwelling consist of?<br>Note: exclude bathrooms and toilets.	Rooms.......... [ ][ ]	
4	How many rooms in your dwelling are used for sleeping?<br>Note: a room may also have another purpose besides as a bedroom.	Rooms for sleeping.......... [ ][ ]	
5	Do you have windows in your household that open easily?	Yes....1<br>No....2<br>Do not have windows in the household....3	
6	Are you able to ventilate or allow "fresh air" through your household?	Yes....1<br>No....2	
7	For what length of time do the windows stay open every day?	2-4 Hours....1<br>4-6 Hours....2<br>More than 6 hours....3	
8	What is the main source of drinking water for members of your household?	Piped water(tap) in dwelling....1<br>Piped water (tap) in site / yard....2<br>Bottled water....3<br>Water carrier/ tanker....4<br>Rain water tank....5<br>Borehole / well / spring....6<br>Dam / river / stream....7<br>Public / communal tap....8<br>Other (Specify)....9	➢ 10<br>➢ 10<br>➢ 10
9	How long does it take you to go there, get water, and come back?	Minutes.......... [ ][ ][ ]	
10	What is this household's main source of water for household use (other than for drinking)?	Regional / local water scheme (operated by municipality or other services provider)....1<br>Borehole....2<br>Spring....3<br>Rain-water tank....4<br>Dam / pool/ stagnant water....5<br>River / stream....6<br>Water vendor....7<br>Water tanker....8<br>Other (Specify)....9	

SECTION E	HOUSING, HOUSEHOLD GOODS AND SERVICES		
NO.	QUESTIONS AND FILTERS	CODING CATEGORIES	SKIP
11	What kind of toilet facilities does your household have?	Flush toilet (connected to sewerage system) ... 1<br>Flush toilet (with septic tank) ... 2<br>Chemical toilet ... 3<br>Pit toilet with ventilation (VIP) ... 4<br>Pit toilet without ventilation ... 5<br>Bucket toilet ... 6<br>Other (Specify) ... 7<br>None ... 8	➢ 13
12	Do you share this toilet with other households	Yes ... 1<br>No ... 2	
13	What is this household's <u>main</u> source of energy for <u>cooking</u> purposes?	Electricity ... 1<br>Coal ... 2<br>Wood ... 3<br>Gas ... 4<br>Paraffin ... 5<br>Animal dung ... 6<br>Solar ... 7<br>Other (Specify) ... 8<br>None ... 9	
14	What is this household's <u>main</u> source of energy for <u>heating</u> purposes?	Electricity ... 1<br>Coal ... 2<br>Wood ... 3<br>Gas ... 4<br>Paraffin ... 5<br>Animal dung ... 6<br>Solar ... 7<br>Other (Specify) ... 8<br>None ... 9	
15	What is this household's <u>main</u> source of energy for <u>lighting</u> purposes?	Electricity ... 1<br>Gas ... 2<br>Paraffin ... 3<br>Candles ... 4<br>Solar ... 5<br>Other (Specify) ... 6<br>None ... 7	
16	How is refuse or rubbish in this household mainly disposed of?	Removed by local authorities at least once a week ... 1<br>Removed by local authorities less than once a week ... 2<br>Communal refuse dump ... 3<br>Own refuse dump ... 4<br>Burn refuse / rubbish ... 5<br>No rubbish disposal ... 6<br>Other (Specify) ... 7	

SECTION E	HOUSING, HOUSEHOLD GOODS AND SERVICES		
NO.	QUESTIONS AND FILTERS	CODING CATEGORIES	SKIP
17	Does this household own any of the following in working order?<br><br>Read options<br>Enter a response for each option	YES NO<br>Fridge…… 1 2<br>Electric / gas stove…… 1 2<br>Vacuum cleaner (Hoover)…… 1 2<br>Washing machine…… 1 2<br>Computer…… 1 2<br>DSTV…… 1 2<br>DVD Player…… 1 2<br>Motorcar……1 2<br>Television……1 2<br>Radio……1 2<br>Telephone (landline)……1 2<br>Cell phone……1 2<br>Mail box / Bag……1 2<br>Mail delivery at home……1 2	
18	If you don't have a telephone, where do members of your household mostly use a telephone if they need one?	At a neighbour nearby……1<br>At a public telephone nearby …… 2<br>At another location nearby …… 3<br>Somewhere else not nearby… …… 4<br>Nowhere …… 5<br>Other (Specify)…… 6	
19	How does this household mainly access the internet?	From home ……1<br>From cell phone……2<br>From work……3<br>From elsewhere……4<br>No access to the internet……5	
20	Does this household engage in any agricultural (vegetable gardening) activities?	Yes……1<br>No ……2	➢ 23
21	What kind of agricultural activities (vegetable gardening) is this household involved in?<br><br>Multiple responses possible<br>Read options	Livestock production (cattle, goats, sheep, pigs, etc) ……1<br>Poultry production (chicken, ducks, geese, guinea fowl, ostrich, etc) ……2<br>Vegetable production……3<br>Production of other crops (grains, fruit, etc)……4<br>Fodder grazing / pasture / grass for animals……5<br>Other (Specify)……6<br>None ……7	
22	Where does the household operate its agricultural activities (vegetable gardening)?<br><br>Read options	Farm land……1<br>Back yard or school……2<br>Communal or tribal land ……3<br>Other (Specify)……4<br>Not applicable ……5	
23	How far do you live from the nearest health clinic or hospital?	0-10 Kilometres……1<br>11-20 Kilometres……2<br>21-30 Kilometres……3<br>More than 30 Kilometres ……4	
24	How far do you live from the nearest primary school?	0-10 Kilometres……1<br>11-20 Kilometres……2<br>21-30 Kilometres……3<br>More than 30 Kilometres ……4	

SECTION E	HOUSING, HOUSEHOLD GOODS AND SERVICES		
NO.	QUESTIONS AND FILTERS	CODING CATEGORIES	SKIP
25	How far do you live from the nearest high school?	0-10 Kilometres.....1<br>11-20 Kilometres.....2<br>21-30 Kilometres.....3<br>More than 30 Kilometres.....4	

SECTION F	ACCESSIBILITY OF SERVICES		
NO.	QUESTIONS AND FILTERS	CODING CATEGORIES	SKIP
	**I want to ask you about the places and facilities that are in your neighbourhood within easy walking distance (10-15 minutes). I will read you some statements.**<br>**Please tell me how strongly you agree or disagree with each.**		
1	Many shops, stalls, markets or other places to buy things I need are within easy walking distance of my home.<br><br>Read options	Strongly disagree.....1<br>Somewhat disagree.....2<br>Don't know.....3<br>Somewhat agree.....4<br>Strongly agree.....5	
2	It is within a 10-15 minute walk to a public transport stop (i.e. bus stop, train station or taxi rank) from my home<br><br>Read options	Strongly disagree.....1<br>Somewhat disagree.....2<br>Don't know.....3<br>Somewhat agree.....4<br>Strongly agree.....5	
3	There are pavements on most of the streets in my neighbourhood.<br><br>Read options	Strongly disagree.....1<br>Somewhat disagree.....2<br>Don't know.....3<br>Somewhat agree.....4<br>Strongly agree.....5	
4	There are facilities to cycle in or near my neighbourhood, such as special lanes, separate paths or paths for shared use between bicycles and persons walking.<br><br>Read options	Strongly disagree.....1<br>Somewhat disagree.....2<br>Don't know.....3<br>Somewhat agree.....4<br>Strongly agree.....5	
5	My neighbourhood has several free or affordable recreational facilities, such as parks, walking paths, bike paths, community centres, playgrounds, public swimming pools, etc.<br><br>Read options	Strongly disagree.....1<br>Somewhat disagree.....2<br>Don't know.....3<br>Somewhat agree.....4<br>Strongly agree.....5	
6	The crime rate in my neighbourhood makes it unsafe to go for a walk at night.<br><br>Read options	Strongly disagree.....1<br>Somewhat disagree.....2<br>Don't know.....3<br>Somewhat agree.....4<br>Strongly agree.....5	

SECTION G	COST OF LIVING		
**NO.**	**QUESTIONS AND FILTERS**	**CODING CATEGORIES**	**SKIP**
1	The cost of living is a concern for many families. Can you tell me which option best describes your household's situation?<br><br>Read options	Not enough money for basic things like food and clothes .... 1<br>Money for food and clothes, but short on many other things .... 2<br>We have most of the important things, but few luxury goods............ 3<br>Money for extra things such as holidays and luxury goods................ 4	
2	What monthly income level do you consider to be minimal for your household, i.e. your household could not make ends meet with less?	R	
3	Is the total monthly income of your household higher, lower or more or less the same as this figure?<br><br>Read options	Much higher.. 1<br>Higher .... 2<br>More or less the same.. 3<br>Lower.. 4<br>Much lower .... 5<br>Don't know... 6	
4	Thinking of the next 12 months, do you expect your financial situation to……………………..?<br><br>Read options	Improve.. 1<br>Stay about the same .... 2<br>To get worse.. 3	

4 INSTRUCTION TO INTERVIEWER	Please explain that the information will be kept confidential

Could you please share with us a contact telephone number that we can use to reach you?

Name	Telephone Number									

Could you also please share with us a contact telephone number of a neighbour or a relative who we can contact if we cannot reach you?

Name	Telephone Number									

Thank you for providing information about this household.

Now we will be interviewing individual persons residing in this household

5 INSTRUCTION TO INTERVIEWER	Record the time at the end of the interview	H	H	:	M	M

REFUSAL PARTICULARS (if applicable)

NO.	QUESTIONS AND FILTERS	CODING CATEGORIES
1	At what point did the respondents refuse to take part in the survey?	At the gate or door .... 1<br>After explanation of the survey .... 2<br>After identifying the respondent .... 3<br>During the household interview .... 4<br>Other (Specify) 5
2	What was the reason for the refusal?	Too busy to grant interview .... 1<br>Not available now .... 2<br>Too late in the evening .... 3<br>Don't participate in surveys .... 4<br>Objected to the topic of the survey .... 5<br>Objected to providing information on household members .... 6<br>Do not allow strangers on property .... 7<br>Participated in the recent population census………………….. 8<br>Other (Specify) 9

APPOINTED FIELD CHECKER	TEAM LEADER	OFFICE CHECKER
NAME	NAME	NAME
NUMBER	NUMBER	NUMBER

LAST TWO DIGITS OF THE VISITING POINT QUESTIONNAIRE NUMBER				NUMBER OF HOUSEHOLDS AT THE VISITING POINT																							
				1	2	3	4	5	6	7	8	9	10	11	12	13	14	15	16	17	18	19	20	21	22	23	24
1	26	51	76	1	1	1	3	2	4	1	3	5	8	6	5	12	10	1	6	8	7	19	19	13	21	13	24
2	27	52	77	1	2	3	4	3	1	2	2	3	4	8	3	7	2	5	14	4	15	4	8	6	16	14	22
3	28	53	78	1	1	2	1	4	2	7	6	9	3	5	11	2	1	3	11	7	10	16	16	10	5	2	2
4	29	54	79	1	2	3	2	1	3	5	8	6	2	4	2	4	8	11	10	16	6	9	10	15	11	12	11
5	30	55	80	1	1	1	4	5	6	3	5	7	5	9	8	13	3	2	13	5	18	1	4	1	20	11	5
6	31	56	81	1	2	2	2	3	5	6	7	8	7	1	4	9	14	8	2	17	17	14	12	14	22	10	3
7	32	57	82	1	2	1	1	4	1	4	1	4	6	3	6	5	7	13	9	2	3	13	14	8	2	7	20
8	33	58	83	1	1	2	3	2	5	1	4	2	1	7	10	6	5	4	15	10	5	2	13	4	17	5	17
9	34	59	84	1	1	3	2	5	6	2	2	1	9	10	1	10	4	6	6	1	9	10	1	5	6	9	1
10	35	60	85	1	2	2	4	1	3	3	6	9	10	11	12	3	9	15	7	8	11	6	3	9	4	3	10
11	36	61	86	1	1	1	3	1	4	5	3	1	6	2	9	13	11	14	4	11	4	15	15	17	1	1	23
12	37	62	87	1	2	3	1	3	2	7	5	6	5	7	7	8	6	10	3	3	1	12	20	7	13	22	12
13	38	63	88	1	1	2	1	5	3	6	4	3	4	6	2	11	13	12	1	15	8	7	2	12	15	21	13
14	39	64	89	1	2	3	2	4	1	4	7	8	2	5	6	11	12	9	16	13	16	11	18	18	14	16	18
15	40	65	90	1	2	1	4	2	4	3	8	7	7	11	1	3	5	7	12	14	13	8	17	20	19	20	19
16	41	66	91	1	1	3	3	1	6	5	1	5	9	10	3	2	11	13	8	12	12	5	6	21	8	8	4
17	42	67	92	1	1	2	2	3	4	2	6	2	3	2	12	5	2	10	13	5	8	18	9	16	10	17	16
18	43	68	93	1	2	1	4	2	6	4	1	4	8	9	10	7	9	3	12	12	9	7	20	19	9	19	21
19	44	69	94	1	2	2	1	3	5	2	8	9	10	4	9	8	13	1	1	14	10	19	10	11	18	15	7
20	45	70	95	1	1	3	2	5	4	1	3	8	1	3	8	6	6	9	5	7	13	4	15	1	7	22	15
21	46	71	96	1	1	1	2	5	1	7	2	3	2	1	11	4	7	5	3	2	1	3	12	18	5	19	14
22	47	72	97	1	2	1	3	1	3	2	6	2	1	8	7	1	4	2	11	8	2	17	4	17	21	16	3
23	48	73	98	1	2	3	4	2	2	6	7	7	8	3	4	9	3	6	2	11	11	16	2	8	11	23	6
24	49	74	99	1	1	2	1	4	6	3	5	5	3	1	5	13	1	14	8	14	6	15	9	14	3	6	9
25	50	75	100	1	1	2	3	3	2	4	6	4	7	5	3	12	12	12	4	6	2	17	11	2	12	4	8
