# Supplementary material for: Predictors of knowledge about tuberculosis: results from SANHANES I, a national, cross-sectional household survey in South Africa
Source: BMC Public Health. 2016 Mar 18;16:276. doi: 10.1186/s12889-016-2951-y (PMC4797251; doi:10.1186/s12889-016-2951-y)
Supplement: Additional file 2: — Adult Questionnaire: 15 years and older. (PDF 294 kb) [file 12889_2016_2951_MOESM2_ESM.pdf]

BARCODE

Individual Questionnaire Number: ______________________

THE SOUTH AFRICAN NATIONAL HEALTH AND NUTRITION EXAMINATION SURVEY, 2011/2012

(SANHANES-1)

Adult Questionnaire: 15 years and older

1. Geographic and Interview Particulars

Province	
Enumerator area (EA)	
Visiting point Number (taken from the EA map)	
Visiting point questionnaire number	
**Person number of respondent**	

2. Interview Details

	Year	Month	Day	Time code	Response code
First visit	2012				
Second visit	2012				
Third visit	2012				
Fourth visit	2012				
				**Final response code**	

Time codes

1 = Morning till 12h00
2 = 12h01-15h00
3 = 15h01-18h00
4 = 18h01-21h00
5 = 21h01 and later

Response codes

1 = Interview completed and clinic appointment made
2 = Interview completed but NO clinic appointment made
3 = Partly completed
4 = Appointment made for interview
5 = Selected respondent not at home
6 = Refusal by respondent / parent / guardian
7 = Other (Specify) ....

**Fieldworker**	**Name**	
	**Staff number**	
**Interview starting time**		H H : M M

NAME / NICKNAME OF THIS RESPONDENT ______________________________

To ensure that we interview the correct person during the follow up survey, would you mind giving us your identity number / passport number?

South African Identity number / Passport number													

[1] INSTRUCTION TO INTERVIEWER	Record the answers by circling a response or by writing the answer in the space provided. Please note that only one response is allowed per question unless another instruction is given. Please note that coding categories should NOT be read to the participant unless another instruction is given

[2] INSTRUCTION TO INTERVIEWER	Tick the appropriate consent forms completed for this respondent

CONSENT FORM PERSON 18 YEARS AND OLDER…………… ☐

PARENT / GUARDIAN CONSENT FORM 0 TO 17………………….. ☐

ASSENT FORM CHILDREN AGED 15 TO 17……………………… ☐

HOUSEHOLD CONSENT TO PARTICIPATE IN THE COHORT…. ☐

SECTION A BIOGRAPHIC DETAILS OF THE RESPONDENT

NO.	QUESTIONS AND FILTERS	CODING CATEGORIES	SKIP
1	How old were you at your last birthday?	Age in completed years ☐☐	
2	Birth date of the respondent	Birth Date D D M M Y Y Y Y	
3	Is the respondent a male or female?	Male 1<br>Female 2	
4	Race of the respondent?	African 1<br>White 2<br>Coloured 3<br>Indian/Asian 4<br>Other (Specify) 5	
5	What is your nationality?	South African Citizen 1<br>Non - citizen (permanent resident) 2<br>Non-citizen (refugee) 3<br>Other (Specify) 4	
6	How would you describe your present employment situation?	Housewife, homemaker, not looking for work 1<br>Housewife, homemaker, looking for work 2<br>Unemployed, looking for work 3<br>Unemployed, not looking for work 4<br>Work in informal sector, not looking for permanent work 5<br>Sick disabled and unable to work 6<br>Student / pupil/ learner 7<br>Self-employed – Full time (40 hours or more per week) 8<br>Self-employed – Part time (less than 40 hours per week) 9<br>Employed full time (40 hours or more per week) 10<br>Employed part time (less than 40 hours per week) 11<br>Employed (seasonal work) 12<br>Retired 13<br>Other (Specify) 14	

SECTION B-8	PREGNANCY		
NO.	QUESTIONS AND FILTERS	CODING CATEGORIES	SKIP
6	During which trimester did you smoke tobacco or use tobacco products?<br>Multiple responses possible	During the 1st trimester....1<br>During the 2nd trimester....2<br>During the 3rd trimester....3	
7	During pregnancy, did you ever have a drink containing alcohol?	Yes....1<br>No....2<br>Don't know....3	 ➢C<br>➢C
8	During pregnancy, how many drinks containing alcohol did you have per day?	1 or 2 per week....1<br>3 or 4 per week....2<br>5 or 6 per week....3<br>1 or 2 per day....4<br>3 or 4 per day....5<br>5 or 6 per day....6<br>7 - 9 per day....7<br>10 or more per day....8	
9	During which trimester did you have drink(s) containing alcohol?<br>Multiple responses possible	During the 1st trimester....1<br>During the 2nd trimester....2<br>During the 3rd trimester....3	

SECTION C — TUBERCULOSIS

THE FOLLOWING QUESTIONS ARE ABOUT TUBERCULOSIS (TB)

SECTION C-1	TB: KNOWLEDGE AND AWARENESS		
NO.	QUESTIONS AND FILTERS	CODING CATEGORIES	SKIP
1	Where did you first learn about tuberculosis or TB?<br>Multiple responses possible	Newspapers and magazines....1<br>Radio....2<br>TV....3<br>Billboards....4<br>Brochures, posters and other printed materials....5<br>Health workers....6<br>Family, friends, neighbours or colleagues....7<br>Religious leaders....8<br>Teachers....9<br>Other (Specify)....10	
2	In your opinion, how serious a disease is TB?	Very serious....1<br>Somewhat serious....2<br>Not very serious....3	
3	What are the signs and symptoms of TB?<br>Multiple responses possible	Rash....1<br>Cough....2<br>Cough that lasts longer than 3 weeks....3<br>Coughing up blood....4<br>Severe headache....5<br>Nausea....6<br>Weight loss....7<br>Fever....8<br>Fever without clear cause that lasts more than 7 days....9<br>Chest pain....10<br>Shortness of breath....11<br>Ongoing fatigue....12<br>Don't know....13<br>Other (Specify)....14	

SECTION C-1 TB: KNOWLEDGE AND AWARENESS

NO.	QUESTIONS AND FILTERS	CODING CATEGORIES	SKIP
4	How can a person get TB?<br><br>Multiple responses possible	Through handshakes....1<br>Through the air when a person sneezes or coughs....2<br>Through sharing dishes....3<br>Through eating from the same plate....4<br>Through touching items in public places (doorknobs, handles in transportation, etc)....5<br>Don't know....6<br>Other (Specify)....7	
5	How can a person prevent getting TB?<br><br>Multiple responses possible	Avoid shaking hands....1<br>Covering the mouth when coughing or sneezing....2<br>Avoid sharing dishes....3<br>Washing hands after touching items in public places....4<br>Closing windows at home....5<br>Through good nutrition....6<br>By praying....7<br>Don't know....8<br>Other (Specify)....9	
6	In your opinion, who could get TB?<br><br>Multiple responses possible	Anybody....1<br>Poor people....2<br>Homeless people....3<br>Alcoholics....4<br>Drug users....5<br>People living with HIV/AIDS....6<br>People who have been in prison....7<br>Don't know....8<br>Other (Specify)....9	
7	Can TB be cured?	Yes....1<br>No....2<br>Don't know....3	 ->9<br>->9
8	How can someone with TB be cured?<br><br>Multiple responses possible	Herbal remedies....1<br>Home rest without medicine....2<br>Praying....3<br>Specific drugs given by the health centre....4<br>Directly Observed Treatment support (DOTS)....5<br>Don't know....6<br>Other (Specify)....7	
9	Are people with TB also HIV positive?	Yes....1<br>No....2<br>Don't know....3	
10	Should people with TB also be tested for HIV?	Yes....1<br>No....2<br>Don't know....3	

SECTION C-2 ATTRIBUTION OF TB

NO.	QUESTIONS AND FILTERS	CODING CATEGORIES	SKIP
1	Have you ever been diagnosed with TB?	Yes....1<br>No....2	 ->C-3
2	Please specify how old you were at the time?<br>If more than once, record the age at the last diagnosis	Age in years.... |_|_|	
3	What do you believe caused you to become sick with TB?	....<br>....	
4	Did you go to a traditional healer when you were sick with TB?	Yes....1<br>No....2	

SECTION C-2	ATTRIBUTION OF TB		
NO.	QUESTIONS AND FILTERS	CODING CATEGORIES	SKIP
5	Did you get treatment for TB when you had TB?	Yes....1<br>No....2	 -➢9
6	Did you complete your treatment for TB?	Yes....1<br>No....2	
7	Did you ever miss your treatment?	Yes....1<br>No....2	 -➢9
8	Why did you miss your treatment?<br><br>Multiple responses possible<br><br>Write the responses in the space provided below, given to you by the participant and after the interview is completed, circle the coding categories from the list on the right that is the nearest to the responses given by the participant<br><br>__________<br>__________<br>__________<br>__________<br>__________<br>__________<br>__________	Personal - Feeling<br>I felt better....1<br>I felt better and gave up....2<br>I was depressed....3<br>I was not getting any better / felt worse....4<br>I was too busy with family obligations....5<br>I didn't like being separated from my home/ family....6<br>Financial reasons<br>I had to look for a job or money....7<br>I couldn't afford transport to the clinic....8<br>School / Employment<br>I had to go back to school....9<br>Employer refused permission to go to the clinic....10<br>I didn't want to lose my job....11<br>I couldn't get enough time off from work....12<br>Clinic / Health Services<br>The clinic was too far....13<br>The clinic ran out of medicine / medicine shortage....14<br>The doctors/ nurses treated me badly....15<br>Didn't get enough support / no DOTS supporter....16<br>DOTS supporter of the opposite sex....17<br>Medication<br>I was taking pills for too long.... 18<br>I was taking too many pills....19<br>The pills gave me side effects....20<br>Other<br>A traditional healer told me to stop....21<br>A friend / family member told me to stop....22<br>I forgot to come in for TB treatment....23<br>Other (Specify)....24<br>....................	
9	Does your workplace offer TB treatment support?	Yes....1<br>No....2<br>Don't know....3<br>Don't work....4	

SECTION C-3	TB: ATTITUDES AND CARE SEEKING BEHAVIOUR		
NO.	QUESTIONS AND FILTERS	CODING CATEGORIES	SKIP
1	What would be your reaction if you found out that you have TB?<br><br>Multiple responses possible	Fear....1<br>Surprise....2<br>Shame....3<br>Embarrassment....4<br>Sadness or hopelessness....5<br>Other (Specify)....6	
2	Who would you talk to about your illness if you had TB?<br><br>Multiple responses possible	Doctor or other medical worker....1<br>Spouse....2<br>Parent....3<br>Children....4<br>Other family member....5<br>Close friend....6<br>No one....7<br>Other (Specify)....8	

SECTION C-3	TB: ATTITUDES AND CARE SEEKING BEHAVIOUR		
NO.	QUESTIONS AND FILTERS	CODING CATEGORIES	SKIP
3	What would you do if you thought you had symptoms of TB?<br><br>Multiple responses possible	Go to a private hospital ....1<br>Go to a private clinic....2<br>Go to a private doctor....3<br>Go to a public hospital....4<br>Go to a public clinic ....5<br>Go to a traditional healer ....6<br>Other (Specify) 7	 <br><br><br><br>-➢ 8<br>-➢ 8
4	If you had symptoms of TB, at what point would you go to a hospital/clinic?<br><br>Multiple responses possible	When treatment on my own does not work....1<br>When symptoms that look like TB last for 3-4 weeks ....2<br>When I realise my symptoms might be related to TB ....3<br>Would not go to a hospital / clinic....4	 <br><br>-➢ 8
5	What type of hospital/clinic would you go to?<br><br>Read the options and ask the respondent to choose one answer only	Private hospital....1<br>Private clinic ....2<br>Private doctor ....3<br>Public hospital ....4<br>Public clinic....5	-➢ 6<br>-➢ 6<br>-➢ 6<br>-➢ 7<br>-➢ 7
6	Why you would choose a **private** hospital/clinic/doctor and not a public hospital/clinic?<br><br>Multiple responses possible	Quality of service<br>Adequate ....1<br>Inadequate ....2<br>Cost<br>Have health insurance / medical aid ....3<br>Do not have health insurance / medical aid ....4<br>Proximity<br>Near to where I live....5<br>Far from where I live....6<br>Waiting times to be treated<br>Shorter waiting times....7<br>Longer waiting times....8<br>Waiting times for follow up<br>Shorter waiting times....9<br>Longer waiting times....10<br>Physical and medical facilities<br>Adequate ....11<br>Inadequate ....12<br>Other (Specify) 13	-➢ 9
7	Why you would choose a **public** hospital/clinic and not a private hospital/clinic/doctor?<br><br>Multiple responses possible	Quality of service<br>Adequate ....1<br>Inadequate ....2<br>Cost<br>Have health insurance / medical aid ....3<br>Do not have health insurance / medical aid ....4<br>Proximity<br>Near to where I live....5<br>Far from where I live....6<br>Waiting times to be treated<br>Shorter waiting times....7<br>Longer waiting times....8<br>Waiting times for follow up<br>Shorter waiting times....9<br>Longer waiting times....10<br>Physical and medical facilities<br>Adequate ....11<br>Inadequate ....12<br>Other (Specify) 13	-➢ 9

SECTION C-3	TB: ATTITUDES AND CARE SEEKING BEHAVIOUR		
NO.	QUESTIONS AND FILTERS	CODING CATEGORIES	SKIP
8	What is the reason that you would not go to a hospital/clinic?<br><br>Multiple responses possible	Not sure where to go ....1<br>Cost ....2<br>Difficulties with transport / distance to clinic....3<br>Do not trust medical workers....4<br>Do not like the attitude of medical workers ....5<br>Cannot leave work (overlapping work hours with medical facility working hours)......6<br>Do not want to find out that something is really wrong ....7<br>Other (Specify) 8	
9	How expensive do you think TB diagnosis and treatment is in this country?<br><br>Note the monetary amount	It is free of charge....1<br>It is reasonably priced ....2<br>It is somewhat / moderately expensive ....3<br>It is very expensive....4<br><br>R____________________	

SECTION C-4	TB: ATTITUDES AND STIGMA		
NO.	QUESTIONS AND FILTERS	CODING CATEGORIES	SKIP
1	Which statement is closest to your feeling about people with TB disease?<br><br>Read the options and ask the respondent to choose one answer only	I feel sorry for them but I would like to help them .... 1<br>I feel sorry for them but I tend to stay away from these people.... 2<br>It is their problem and I cannot get TB .... 3<br>I fear them because they may infect me .... 4<br>I have no particular feeling .... 5<br>Other (Specify) 6	
2	In your community, how is a person who has TB usually regarded / treated?<br>Multiple responses possible	Most people reject him or her.... 1<br>Most people are friendly but they generally try to avoid him or her.... 2<br>The community mostly supports him or her .... 3<br>Other (Specify) 4	
3	Should HIV positive people be concerned about TB?	Yes.... 1<br>No 2	 -➢ 5
4	Why?	Person with HIV is more likely to develop TB .... 1<br>Don't know.... 2<br>Other (Specify) 3	
5	Why not?	Person with HIV is not more likely than a person without HIV to develop TB .... 1<br>Don't know.... 2<br>Other (Specify) 3	

SECTION C-5	SOURCES OF TB INFORMATION		
NO.	QUESTIONS AND FILTERS	CODING CATEGORIES	
1	Are you well informed about TB?	Yes....1<br>No 2	
2	Would you like to have more information about TB?	Yes....1<br>No 2	
3	What are the sources of information that you think can most effectively reach people like you with information on TB?<br><br>Read options and respondent to choose the THREE most effective sources	Newspapers and magazines....1<br>Radio ....2<br>TV ....3<br>Billboards....4<br>Brochures, posters and other printed materials....5<br>Health workers....6<br>Family, friends, neighbours and colleagues ....7<br>Religious leaders....8<br>Teachers....9<br>Other (Specify) 10	
