# Supplementary material for: Predictors of knowledge about tuberculosis: results from SANHANES I, a national, cross-sectional household survey in South Africa
Source: BMC Public Health. 2016 Mar 18;16:276. doi: 10.1186/s12889-016-2951-y (PMC4797251; doi:10.1186/s12889-016-2951-y)
Supplement: Additional file 3: — Correct knowledge for the six individual knowledge items by demographic characteristics, TB diagnosis and sources of information on TB. (DOCX 33 kb) [file 12889_2016_2951_MOESM3_ESM.docx]

Appendix 1. Correct knowledge for the six individual knowledge items by demographic characteristics, TB diagnosis and sources of information on TB

|  | **Knowledge on Symptoms^1^** | | **Knowledge on Transmission^2^** | | **Knowledge on Prevention^3^** | | **Knowledge on curability and correct treatment method^4^** | | **Knowledge that TB patients should be tested for HIV^5^** | | **Knowledge that people with HIV are more likely to develop TB^6^** | |  |
| --- | --- | --- | --- | --- | --- | --- | --- | --- | --- | --- | --- | --- | --- |
|  | **%** | **95% CI** | **%** | **95% CI** | **%** | **95% CI** | **%** | **95% CI** | **%** | **95% CI** | **%** | **95% CI** | **n** |
| **Total** | 21.5 | [19.0-24.1] | 63.0 | [59.8-66.1] | 79.0 | [76.9-81.0] | 83.9 | [81.5-86.0] | 83.1 | [81.1-84.9] | 76.1 | [73.1-78.9] | 5945 |
| **Age** |  |  |  |  |  |  |  |  |  |  |  |  |  |
| 18 to 24 | 22.3 | [18.4-26.7] | 65.8 | [60.6-70.5] | 79.7 | [74.9-83.8] | 83.7 | [80.2-86.7] | 82.1 | [78.7-85.2] | 73.5 | [68.5-77.9] | 1168 |
| 25 to 34 | 20.7 | [17.0-25.0] | 65.1 | [60.2-69.7] | 79.9 | [76.4-82.9] | 82.7 | [79.3-85.6] | 86.0 | [83.0-88.6] | 77.0 | [72.9-80.6] | 1405 |
| 35 to 44 | 20.5 | [17.0-24.5] | 61.0 | [56.0-65.9] | 80.2 | [76.9-83.2] | 85.3 | [81.4-88.5] | 83.7 | [79.8-87.0] | 78.0 | [73.7-81.7] | 1283 |
| 45 to 54 | 23.9 | [19.5-29.0] | 61.1 | [55.2-66.7] | 77.3 | [73.9-80.4] | 83.6 | [78.9-87.5] | 80.5 | [76.4-84.1] | 75.5 | [69.9-80.3] | 1218 |
| 55 to 64 | 20.2 | [16.0-25.2] | 61.0 | [55.1-66.6] | 76.1 | [71.7-80.1] | 84.4 | [80.9-87.4] | 80.4 | [75.6-84.4] | 75.5 | [70.7-79.7] | 871 |
| **Sex** |  |  |  |  |  |  |  |  |  |  |  |  |  |
| Female | 22.5 | [19.3-26.1] | 62.5 | [59.0-65.9] | 80.2 | [77.8-82.4] | 85.2 | [82.9-87.2] | 83.2 | [80.8-85.3] | 76.6 | [73.1-79.7] | 3509 |
| Male | 20.3 | [17.4-23.5] | 63.6 | [59.6-67.4] | 77.7 | [74.8-80.4] | 82.4 | [79.2-85.2] | 83.1 | [80.6-85.3] | 75.7 | [72.5-78.6] | 2436 |
| **Race** |  |  |  |  |  |  |  |  |  |  |  |  |  |
| African | 20.4 | [17.8-23.3] | 63.4 | [59.9-66.7] | 79.4 | [76.9-81.6] | 82.9 | [80.3-85.2] | 81.8 | [79.4-83.9] | 73.8 | [70.4-77.0] | 3824 |
| White | 34.1 | [25.5-44.0] | 55.9 | [42.2-68.8] | 78.5 | [70.2-85.0] | 84.9 | [71.8-92.5] | 86.9 | [78.3-92.4] | 81.4 | [67.4-90.2] | 312 |
| Coloured | 16.5 | [12.8-21.0] | 67.7 | [62.9-72.2] | 77.4 | [73.2-81.1] | 90.5 | [87.8-92.6] | 89.2 | [85.4-92.2] | 84.9 | [80.8-88.3] | 1394 |
| Indian | 20.8 | [15.1-28.0] | 62.6 | [52.7-71.5] | 78.3 | [61.2-89.2] | 81.1 | [71.8-87.9] | 82.2 | [75.2-87.6] | 89.6 | [82.0-94.2] | 415 |
| **Urban / Rural** |  |  |  |  |  |  |  |  |  |  |  |  |  |
| Rural | 17.8 | [14.4-21.8] | 63.5 | [59.2-67.5] | 75.9 | [72.8-78.8] | 82.4 | [78.8-85.5] | 79.7 | [76.5-82.7] | 71.3 | [67.6-74.7] | 1850 |
| Urban | 23.2 | [20.1-26.7] | 62.8 | [58.5-67.0] | 80.5 | [77.7-83.1] | 84.6 | [81.4-87.2] | 84.7 | [82.1-87.0] | 78.4 | [74.3-82.1] | 4095 |
| **Completion of high school/equivalent** |  |  |  |  |  |  |  |  |  |  |  |  |  |
| Did not complete high school | 18.7 | [16.1-21.7] | 61.4 | [58.0-64.7] | 77.2 | [74.4-79.7] | 81.5 | [78.8-83.9] | 79.2 | [76.6-81.5] | 72.8 | [69.6-75.7] | 3774 |
| Completed high school (Grade 12 or higher) | 25.1 | [21.6-28.9] | 65.2 | [60.4-69.6] | 81.5 | [78.0-84.5] | 87.0 | [84.1-89.5] | 88.3 | [85.6-90.6] | 80.6 | [75.8-84.6] | 2171 |
| **Employment Status** |  |  |  |  |  |  |  |  |  |  |  |  |  |
| Unemployed | 19.7 | [17.1-22.7] | 61.1 | [57.8-64.2] | 75.7 | [72.8-78.3] | 83.2 | [80.7-85.5] | 80.3 | [78.0-82.4] | 74.3 | [70.8-77.5] | 3183 |
| Employed | 23.2 | [20.0-26.8] | 65.0 | [60.4-69.4] | 82.5 | [79.9-84.7] | 84.5 | [81.1-87.5] | 86.0 | [83.3-88.3] | 78.0 | [74.5-81.2] | 2762 |
| **Annual income** |  |  |  |  |  |  |  |  |  |  |  |  |  |
| No income | 21.0 | [17.8-24.6] | 60.8 | [56.6-64.9] | 76.8 | [73.2-80.0] | 84.7 | [81.3-87.5] | 81.3 | [78.6-83.8] | 75.4 | [71.1-79.2] | 2118 |
| <=R9 600 | 21.0 | [16.0-27.1] | 68.5 | [62.6-73.9] | 75.8 | [70.7-80.3] | 83.2 | [78.8-86.9] | 81.0 | [76.8-84.5] | 75.5 | [70.6-79.8] | 682 |
| R9 601-R38 400 | 18.7 | [15.5-22.4] | 61.9 | [57.1-66.6] | 78.2 | [74.8-81.2] | 80.1 | [76.6-83.3] | 82.4 | [79.4-85.0] | 69.9 | [65.5-73.9] | 1896 |
| >=R38 401 | 25.6 | [21.3-30.4] | 64.8 | [59.1-70.2] | 84.6 | [81.0-87.6] | 87.4 | [81.9-91.4] | 87.4 | [83.3-90.7] | 84.7 | [79.7-88.6] | 1249 |
| **Have you ever been diagnosed with TB** |  |  |  |  |  |  |  |  |  |  |  |  |  |
| No | 21.4 | [18.9-24.2] | 62.6 | [59.2-65.8] | 78.7 | [76.5-80.8] | 83.3 | [80.9-85.5] | 83.2 | [81.1-85.1] | 75.9 | [72.8-78.8] | 5487 |
| Yes | 22.5 | [17.5-28.3] | 69.4 | [62.9-75.3] | 82.9 | [77.4-87.2] | 90.9 | [86.6-93.9] | 81.7 | [76.1-86.3] | 79.2 | [73.0-84.2] | 458 |
| **Source of Knowledge: Where did you first learn about TB?** |  |  |  |  |  |  |  |  |  |  |  |  |  |
| **Newspapers and magazines** |  |  |  |  |  |  |  |  |  |  |  |  |  |
| No | 19.2 | [16.8-21.9] | 65.0 | [61.7-68.1] | 78.2 | [75.8-80.3] | 84.2 | [81.8-86.3] | 82.6 | [80.5-84.5] | 77.0 | [74.0-79.7] | 4568 |
| Yes | 28.9 | [24.6-33.7] | 56.5 | [50.8-62.0] | 81.9 | [76.6-86.2] | 82.8 | [78.0-86.8] | 84.8 | [79.8-88.8] | 73.3 | [66.9-78.8] | 1377 |
| **Radio** |  |  |  |  |  |  |  |  |  |  |  |  |  |
| No | 15.9 | [13.3-18.9] | 64.7 | [61.1-68.1] | 74.7 | [71.8-77.3] | 84.5 | [82.0-86.8] | 81.6 | [78.9-84.0] | 75.8 | [72.5-78.9] | 3096 |
| Yes | 27.2 | [23.7-31.0] | 61.3 | [56.5-65.9] | 83.6 | [80.3-86.4] | 83.2 | [79.8-86.1] | 84.7 | [81.9-87.1] | 76.5 | [72.4-80.1] | 2849 |
| **Television** |  |  |  |  |  |  |  |  |  |  |  |  |  |
| No | 16.2 | [13.7-19.1] | 64.0 | [60.4-67.4] | 73.0 | [70.0-75.9] | 83.3 | [80.6-85.6] | 81.0 | [78.5-83.3] | 74.2 | [70.3-77.7] | 3357 |
| Yes | 27.7 | [24.0-31.7] | 61.9 | [57.1-66.5] | 86.1 | [83.3-88.5] | 84.6 | [80.7-87.8] | 85.6 | [82.8-88.0] | 78.5 | [74.9-81.7] | 2588 |
| **Brochures, pamphlets, other printed material** |  |  |  |  |  |  |  |  |  |  |  |  |  |
| No | 19.9 | [17.4-22.7] | 63.2 | [59.8-66.5] | 77.9 | [75.7-80.0] | 83.7 | [81.3-85.8] | 83.0 | [81.0-84.8] | 75.7 | [72.6-78.6] | 5450 |
| Yes | 37.3 | [29.2-46.2] | 61.2 | [53.8-68.1] | 90.5 | [86.6-93.3] | 86.0 | [78.5-91.2] | 84.6 | [74.8-91.1] | 80.5 | [71.8-87.0] | 495 |
| **Health workers** |  |  |  |  |  |  |  |  |  |  |  |  |  |
| No | 17.0 | [14.5-19.8] | 65.1 | [61.4-68.7] | 76.1 | [73.5-78.6] | 80.9 | [77.9-83.6] | 82.5 | [80.1-84.7] | 73.6 | [69.6-77.3] | 3710 |
| Yes | 28.8 | [25.3-32.5] | 59.6 | [55.3-63.8] | 83.7 | [81.1-86.0] | 88.7 | [85.9-90.9] | 84.1 | [81.2-86.7] | 80.2 | [77.1-83.0] | 2235 |
| **Family, friends, neighbours, colleagues** |  |  |  |  |  |  |  |  |  |  |  |  |  |
| No | 20.3 | [17.9-22.9] | 64.1 | [60.7-67.3] | 80.6 | [78.5-82.6] | 84.0 | [81.4-86.4] | 83.4 | [81.3-85.3] | 76.5 | [73.3-79.3] | 4855 |
| Yes | 26.9 | [21.7-32.8] | 58.3 | [52.2-64.1] | 71.6 | [65.9-76.7] | 83.1 | [77.8-87.4] | 81.8 | [76.8-86.0] | 74.7 | [69.2-79.5] | 1090 |
| **Teachers** |  |  |  |  |  |  |  |  |  |  |  |  |  |
| No | 19.9 | [17.1-23.0] | 63.0 | [59.3-66.7] | 77.7 | [75.3-79.9] | 82.5 | [79.8-85.0] | 82.1 | [79.8-84.1] | 73.3 | [70.0-76.4] | 4437 |
| Yes | 26.4 | [21.8-31.7] | 63.0 | [57.9-67.9] | 83.3 | [79.6-86.4] | 88.1 | [84.9-90.7] | 86.4 | [82.6-89.5] | 85.1 | [81.2-88.3] | 1508 |
| **Other source of information** |  |  |  |  |  |  |  |  |  |  |  |  |  |
| No | 20.7 | [18.4-23.2] | 63.9 | [60.8-66.8] | 79.1 | [77.0-81.1] | 84.4 | [82.1-86.6] | 83.1 | [81.1-84.9] | 76.6 | [73.7-79.3] | 5288 |
| Yes | 27.8 | [21.2-35.5] | 55.9 | [46.1-65.2] | 78.3 | [71.1-84.1] | 79.0 | [72.6-84.3] | 83.1 | [77.1-87.7] | 72 | [64.4-78.4] | 657 |

1. Correctly identified 3 of the 6 essential symptoms of TB (according to WHO standardised definitions)

2. Correctly identified the primary transmission method of TB i.e. through the air when a person sneezes or coughs, without any misconceptions

3. Correctly identified the main prevention method i.e. covering the mouth when coughing or sneezing

4. Correct knowledge that TB is curable and correct knowledge of treatment without misconceptions i.e. that it can be cured by specific drugs given by a health centre or by DOTS

5. Correct knowledge that TB patients should be tested for HIV

6. Correct knowledge that people with HIV are more likely to develop TB
